# Integrative analysis reveals functional and regulatory roles of H3K79me2 in mediating alternative splicing

**DOI:** 10.1186/s13073-018-0538-1

**Published:** 2018-04-17

**Authors:** Tianbao Li, Qi Liu, Nick Garza, Steven Kornblau, Victor X. Jin

**Affiliations:** 1 College of Life Science, Jilin University, Changchun, 130012 China; 2 Department of Molecular Medicine, University of Texas Health, 8403 Floyd Curl, San Antonio, TX 78229 USA; 3 Department of Biomedical Engineering, Johns Hopkins University, Baltimore, MD 21218 USA; 4 Department of Leukemia, UT MD Anderson Cancer Center, Houston, TX 77030 USA

**Keywords:** Alternative Splicing, H3K79me2, DOT1L, AML

## Abstract

**Background:**

Accumulating evidence suggests alternative splicing (AS) is a co-transcriptional splicing process not only controlled by RNA-binding splicing factors, but also mediated by epigenetic regulators, such as chromatin structure, nucleosome density, and histone modification. Aberrant AS plays an important role in regulating various diseases, including cancers.

**Methods:**

In this study, we integrated AS events derived from RNA-seq with H3K79me2 ChIP-seq data across 34 different normal and cancer cell types and found the higher enrichment of H3K79me2 in two AS types, skipping exon (SE) and alternative 3′ splice site (A3SS).

**Results:**

Interestingly, by applying self-organizing map (SOM) clustering, we unveiled two clusters mainly comprised of blood cancer cell types with a strong correlation between H3K79me2 and SE. Remarkably, the expression of transcripts associated with SE was not significantly different from that of those not associated with SE, indicating the involvement of H3K79me2 in splicing has little impact on full mRNA transcription. We further showed that the deletion of DOT1L1, the sole H3K79 methyltransferase, impeded leukemia cell proliferation as well as switched exon skipping to the inclusion isoform in two MLL-rearranged acute myeloid leukemia cell lines. Our data demonstrate H3K79me2 was involved in mediating SE processing, which might in turn influence transformation and disease progression in leukemias.

**Conclusions:**

Collectively, our work for the first time reveals that H3K79me2 plays functional and regulatory roles through a co-transcriptional splicing mechanism.

**Electronic supplementary material:**

The online version of this article (10.1186/s13073-018-0538-1) contains supplementary material, which is available to authorized users.

## Background

Alternative splicing (AS) is a pre-mRNA process mainly controlled by post-transcriptional regulation involving 90% of human multi-exonic coding genes in a variety of tissues and cell types [1–3]. Many studies have highlighted the key role of AS in regulating cellular development and differentiation, and aberrant AS events lead to disease states such as muscular dystrophies and cancers [4–6]. Accumulating evidence further supports a new paradigm that AS is a co-transcriptional splicing process mutually coordinated by transcription and splicing [7–9]. Recent studies further illustrate that splicing is also regulated by epigenetic regulators, including chromatin structure, histone modifications, and CTCF [10, 11]. Dysregulation of some epigenetic components may alter the splicing process, resulting in various types of human diseases [12–15]. For instance, a recent study reported that a mutation of the histone methyl transferase SEDT2 alters AS of several key WNT signaling regulatory genes, resulting in colorectal cancer [16].

Recent genome-wide studies revealed histone marks such as H3K36me3 and H3K79me2 as well as nucleosome positioning were highly enriched within intragenic regions, implicating their regulatory roles in the RNA polymerase II elongation process and exon definition [17–20]. Further studies demonstrated the enrichment levels of histone modifications were correlated not only with transcriptional activity, but also with AS [21–23]. Despite these de novo genome-wide findings, knowledge on the causal and functional roles of histone modifications in AS is limited. In addition, little work has been done on aberrant AS processing in diseases caused by epigenetic defects.

H3K79, located in the globular domain of histone H3, is exposed on the nucleosome surface and then methylated by the sole enzyme DOT1-like histone lysine methyltransferase (DOT1L), a member of the lysine methyltransferase family [24]. This histone methylation typically functions in transcriptional regulation [25, 26], telomeric silencing [27, 28], cell-cycle regulation [29], and DNA damage repair [30–32]. Recent studies revealed a new role for it in regulating AS [33–35]. For example, H3K79me2 is able to recruit chromodomain-containing protein MRG15 and splicing factor PTB1 to influence AS outcomes [36, 37]. In particular, new findings demonstrated its crucial role in transformation as well as disease progression in leukemias [38–40]. DOT1L is frequently involved in chromosomal translocations, with numerous genes creating fusion genes that interfere with its interaction with the elongation complexes, resulting in a loss of function. This is common in the mixed-lineage leukemia (MLL) gene, resulting in aggressive leukemia [41], including 5–10% of adult acute leukemias [42] and 60–80% of infant acute leukemias [43]. These findings have established a foundation for disease-specific epigenetic therapies against acute leukemias.

In a previous study, we found a correlation between H3K79me2 enrichment level and an exon skipping event in GM12878 and K562 cells [20]. However, the common and cell type-specific genomic patterns and correlations between H3K79me2 and various types of splicing events across diverse cell types have not been fully explored. In this study, we integrated AS events derived from RNA-seq with H3K79me2 ChIP-seq data across 34 different normal and cancer cell types, and examine the enrichment of H3K79me2 in five major types of AS events, skipping exon (SE), mutual exclusive exon (MXE), retained intron (RI), alternative 5′-end splice site (A5SS), and alternative 3′-end splice site (A3SS). We attempt to elucidate functional and regulatory roles of H3K79me2 in mediating AS, particularly in MLL-rearranged (MLL-r) acute myeloid leukemia (AML) cells.

## Methods

### Raw data processing

H3K79me2 ChIP-seq and RNA-seq data for a total of 34 various normal and cancer cell lines were collected from the Gene Expression Omnibus (GEO) repository and ENCODE Consortia (Additional file 1: Table S1). Raw sequence reads were aligned against the human genomic sequence (GRCh37) using bowtie2 for ChIP-seq data [44] and TopHat (version 2.0.14) for RNA-seq data [45]. Only uniquely mapped reads were used for further downstream analysis.

### Identification of AS events and H3K79me2 enrichment and peaks

Unique reads from RNA-seq data in bam format are used as input for MISO (The Mixture of Isoforms), which detected AS events based on Bayes factors, filtering criteria, Psi values (Ψ) and confidence intervals [46]. Sashimi plots were generated to illustrate all five types of AS events for visualization. The enrichment of H3K79me2/kb is calculated as the number of reads from H3K79me2 ChIP-seq data in exon skipping gene regions (the exon part of an exon skipping gene plus 50 bp upstream and downstream around exons) per kilobase pair (the length of the exon skipping gene region) normalized by the total number of reads of each dataset. The H3K79me2 peaks were identified by Model-based Analysis of ChIP-Seq version 2 (MACS2) with a q value (minimum false discovery rate (FDR)) of 0.01 [47].

### Self-organizing map clustering

We used self-organizing map (SOM) clustering for dimension reduction for feature extraction associated with exon skipping sites. SOM is a model of two-layer artificial neural networks that maps high dimensional input datasets to a set of nodes arranged in lattice. SOM has two steps: (i) determining a winner node and (ii) updating weighted vectors associated with the winner node and some of its neighboring nodes. According to the enrichment of H3K79me2 for each SE site, the SOM algorithm maps multi-dimensional input vectors to two-dimensional neurons, helping to understand the high-dimensional SE data; the most enriched cluster for each cell is assigned to their cell type. The SOM training was performed using the R package “kohonen”. SOM training parameters and node number optimization were defined on the basis of Xie et al. [48]. Node grouping was based on a hierarchical clustering approach using the hclust function of the “Stats” package of R. The number of clusters was chosen based on homogeneity analyses.

### Cell culture and reagents

Human cell lines MV-4-11, K562, and OCI-LY7 were cultured in Iscove’s modified Dulbecco's medium (Thermo Fisher Scientific) and GM12878, MM.1S, and MOLM-14 cell lines were cultured in RPMI-1640/10% fetal bovine serum (FBS; Invitrogen, Carlsbad, CA, USA) at 37 °C in 5% CO_2_. MOLM-14 and OCI-LY7 cells were purchased from the DSMZ (Deutsche Sammlung von Mikroorganismen und Zellkulturen, Braunschweig, Germany), and GM12878, K562, MM.1S, and MV-4-11 cells were purchased from ATCC (American Type Culture Collection).

### Co-transfection and cell viability assay

siRNAs of DOT1L were purchased from Thermo Fisher Scientific Silencer® Select siRNAs. For transfection of siRNA oligos, cells were seeded in six-cell plates with Lipofectamine® RNAiMAX Transfection Reagent for 48 h.

The Cell Counting Kit-8 method was used to measure cell viability. Cells were seeded in 96-well plates at a density of 3 × 10^3^ cells/ml. The viability of cells was assessed using the CCK8 reagent (Dojindo Laboratories, Japan) according to the manufacturer’s protocols. The absorbance at 450 nm was recorded on a microplate reader.

### RT-PCR and ChIP-qPCR

Total RNAs from cells were extracted using Quick-RNA™ MiniPrep kit (Zymo Research). Then cDNA was prepared using a RevertAid H Minus First Strand cDNA Synthesis Kit (Thermo Fisher Scientific). The PCR primers for amplifying cDNA fragments between the upstream exon and downstream exon of five exon-skipping event sites are described in Additional file 1: Table S2. PCR was performed with NEBNext® High-Fidelity 2X PCR Master Mix (New England Biolabs, UK), and the cycling conditions were 98 °C for 1 min, then 30 cycles of 98 °C for 10 s, 58 °C for 20 s, 72 °C for 30 s. PCR products were visualized on 3% agarose gels.

ChIP-qPCR was performed as described in Zhu et al. [49]. Briefly, crosslinking was performed with 1% formalin and the cells were lysed in SDS buffer. DNA was fragmented by sonication with a Covaris S220. Chromatin immunoprecipitation (ChIP) was performed using an antibody to the H3K79me2 modification (Abcam, ab3594). Quantification of ChIP-DNA analysis was performed with the LightCycler® 480 SYBR Green I Masteron and LightCycler® 480 System Sequence Detection System (Roche Applied Science) using GAPDH for normalization with primers listed in Additional file 1**:** Table S3.

## Results

### Identification of the AS events across 34 normal and cancer cell types

**Fig. 1:**
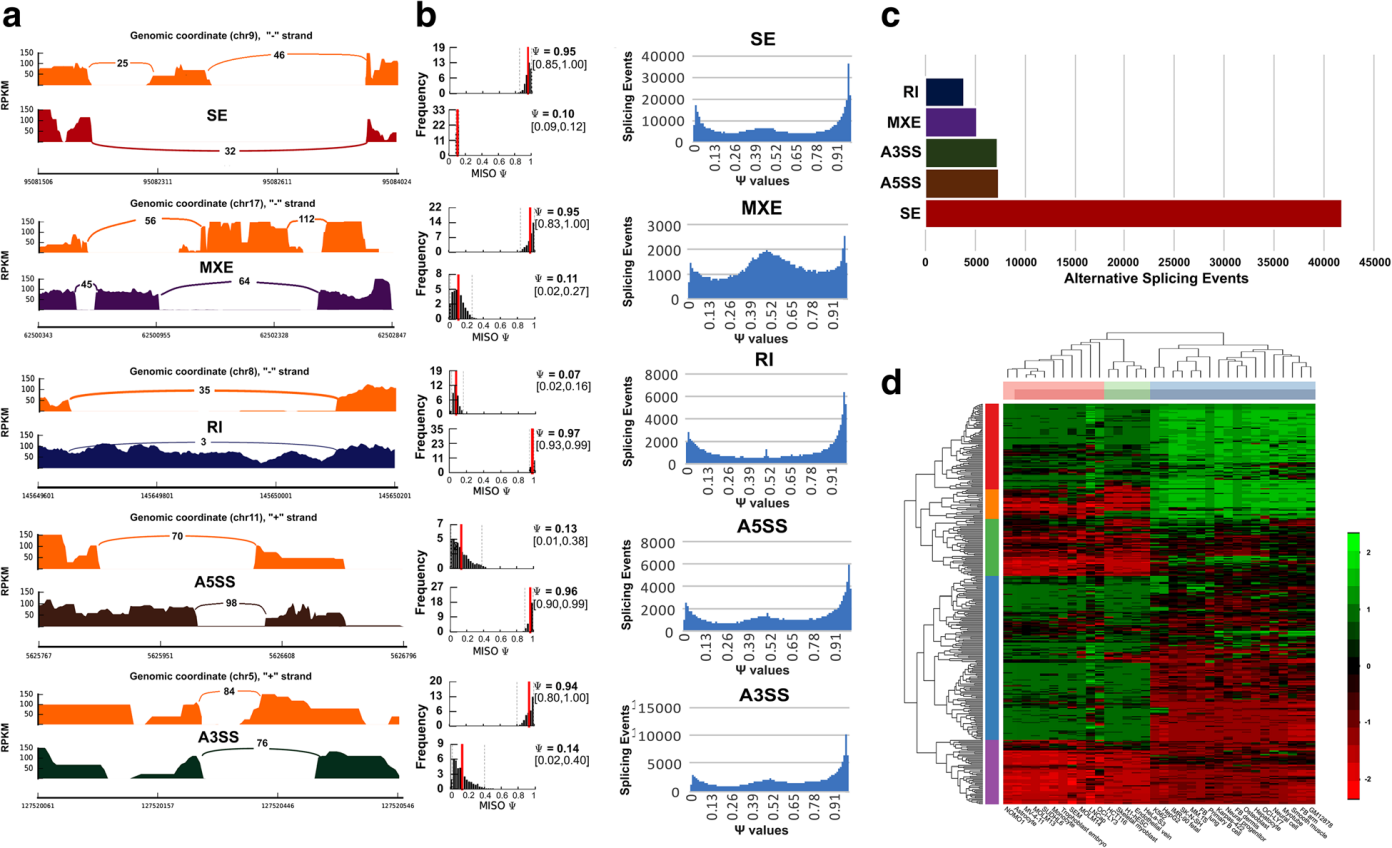
Alternative splicing event detection across 34 normal and cancer cell types. **a** Sashimi plots visualizing a specific splice site for each of five major types of AS events. The constitutive splicing isoform is shown in the *upper track* in *yellow* and the alternative splicing isoform is shown in the *lower track*. Percentage spliced in (ψ) value is shown on the *right side*. *SE* skipped exon, *MXE* mutual exclusive exon, *RI* retained intron, *A5SS* alternative 5′-end splice site, *A3SS* alternative 3′-end splice site. **b** The distribution of ψ-value in five types of AS events identified by the MISO tool. Cutoff values are 0.2 for SE and A3SS and 0.8 for RI and A5SS; for MXE, ψ values in the range 0–0.2 and 0.8–1 were used for two exons mutually exclusive to each other. **c** Total number of AS events for each of five types at the defined ψ value cutoffs. **d** Five types of AS events clustered in 34 cell types showing the difference between normal and cancer cell types. *FB* fibroblast

We obtained both RNA-seq and H3K79me2 ChIP-seq data for a total of 34 different cell types with 18 normal and 16 cancer cell types from the GEO repository and ENCODE Consortia (Additional file 1**:** Table S1). Using the MISO tool and an annotated AS database [46], we first identified exon junction reads, calculated the ψ-value (Psi, percent splice in) for the number of reads aligned to splice junctions vs target exons (Additional file 2**:** Figure S1), and finally determined the specific predominant isoform for each of five major types of AS events: SE, MXE, RI, A5SS, A3SS (Fig. 1a). As demonstrated in Fig. 1b for the ψ-value distribution, ψ-value ≤ 0.2 was used to determine the predominant splicing isoform for SE and A3SS, but ψ-value ≥ 0.8 was used for RI and A5SS; however, either ψ-value was used for MXE. Consequently, we identified a total of 41,840 SE, 5228 MXE, 3909 RI, 7386 A3SS, and 7303 A5SS events for all 34 cell types (Fig. 1c), with SE clearly the major splicing event. We applied unsupervised clustering on all AS events to illustrate the difference among samples. Among the three types of cell clusters, most normal cell types, such as fibroblast, myotube, GM12878, and others, showed distinct AS event patterns compared with cancer or blood cancer cell types **(**Fig. 1d). The numbers of identified AS events were quite diverse in terms of genomic locations and among different cell types, 330–3035 for SE, 0–290 for MXE, 0–249 for RI, 112–301 for A5SS, and 0–345 for A3SS events, respectively (Additional file 2**:** Figure S2 and Additional file 1**:** Table S4).

### Characterization of H3K79me2 enrichment around splice sites

**Fig. 2:**
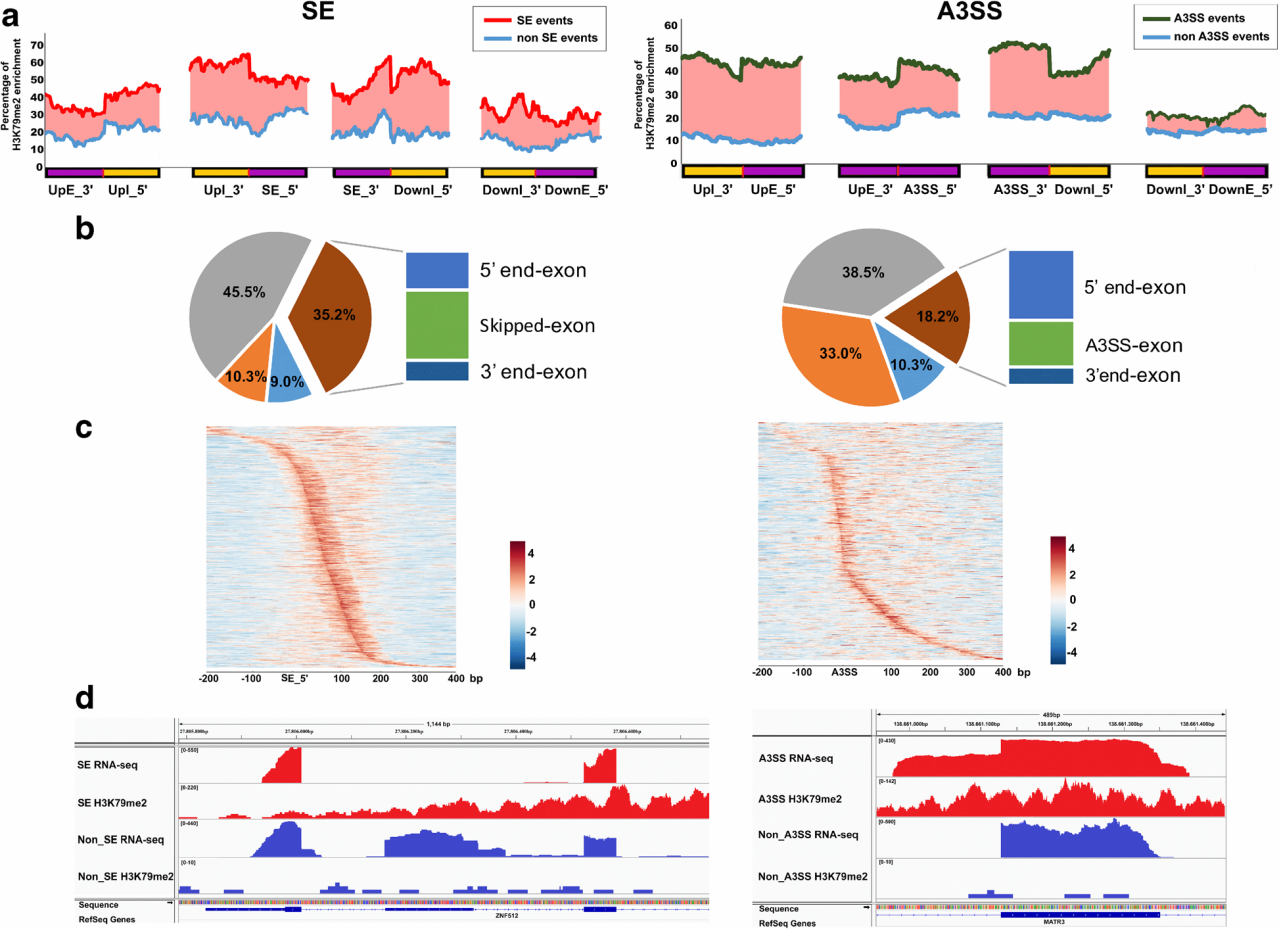
H3K79me2 enrichment ChIP-seq data around splice sites of SE and A3SS. **a** Enrichment plots of H3K79me2 showing the comparison between AS sites and random non-AS sites. *Red*/*green lines* show the average H3K79me2 enrichment of AS events and *blue lines* show the average H3K79me2 enrichment in random non-AS sites, while the *pink area* highlights the discrepancy in enrichment between AS and non-AS sites. **b** Percentage of AS sites enriched with different levels of the H3K79me2 mark after comparison with non-AS sites (*left*) as well as of AS sites with identified H3K79me2 peaks located in alternative sites or junction elements (*right*). **c** Peak patterns identified from H3K79me2 peaks in AS events. Each splicing event with a H3K79me2 peak was aligned by its splicing exon start site, and the density of read occupancy around each event (200 bp upstream and 400 bp downstream) was normalized by a *z*-score method. **d** Two typical examples of genes with SE events: ZNF512 with a specific SE event in GM12878 cells vs non-SE in primary B cells (*left*); and MATR3 with a A3SS event in skeletal muscle myoblast cells vs non-A3SS in arm fibroblast cells (*right*)

Our previous data integration revealed strong enrichment of H3K79me2 at exon skipping sites in GM12878 and K562 cells [20]. To extend this observation, we set out to comprehensively characterize H3K79me2 enrichment with each of the five types of AS events. We first examined the average H3K79me2 enrichment for each AS event for the combined set of all 34 cell types. We were particularly interested in understanding the enrichment at the alternative and junction sites of four discrete genomic regions, including 50 bp around the 5′-end of the splice site, 50 bp around the 3′-end of the splice site, 50 bp around the 3′-end of the upstream exon, and 50 bp around the 5′-end of the downstream exon. We also selected a set of non-AS sites randomly from exons and genes without any AS events as a control. Interestingly, we found only two AS event types, SE and A3SS, were highly enriched with H3K79me2 in comparison to non-splice sites (Fig. 2a). For SE, skipping and junction sites exhibited 118 and 64% higher levels of H3K79me2, respectively, than these random non-skipping sites, and for A3SS, alternative 3′ splice sites and the 3′-end of the upstream exon showed dramatic 187 and 367% increases in enrichment, respectively, but only a 21.5% increase for the 5′-end of the downstream exon. We noted that we did not observe any enrichment of H3K79me2 in the other three splicing events (Additional file 2**:** Figure S3). A close examination of the distribution of H3K79me2 at SE sites showed a diversity of its enrichment levels in each individual cell type (Additional file 2: Figure S4). Further, we identified 33,765 (80.7%) of 41,840 SE sites with higher H3K79me2 enrichment, 10.3% with no significant difference, and 9.0% with decreased enrichment relative to the average H3K79me2 enrichment at non-ES sites. Remarkably, 35.2% of these have an enriched H3K79me2 peak called by MACS2. For A3SS, the numbers were 56.7% (4141 of 7303), 33.0%, and 10.3% with higher, the same, and lower levels of H3K79me2 enrichment compared to non-A3SS sites (Fig. 2b and Additional file 2**:** Figure S3). We further looked into the AS events with H3K79me2 peaks around the skipped exons and A3SS event start sites. The density plot of the raw read enrichment for each event by z-score normalization within a range of 200 bp upstream and 400 bp downstream showed clear H3K79me2 enrichment around exon junction sites toward the skipped exon in SE events and higher H3K79me2 enrichment around the A3SS event start sites (Fig. 2c). We visually illustrate two examples of RNA-seq and H3K79me2 ChIP-seq data in Fig. 2d, a specific SE event in the ZNF512 gene in GM12878 cells vs non-SE in primary B cells and a A3SS event in the MATR3 gene in skeletal muscle myoblast cells vs non-A3SS in arm fibroblast cells.

### SOM clustering for SE sites across 34 different cell types

**Fig. 3:**
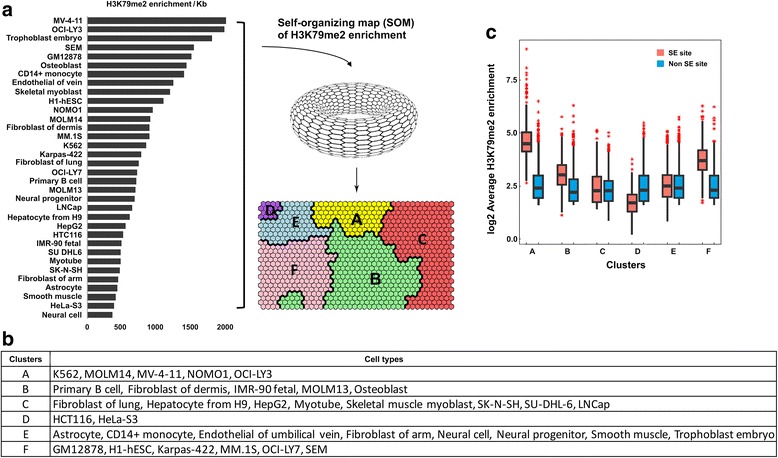
SOM clustering of H3K79me2 enrichment across 34 different cell types. **a** Enrichment data for 7017 genes associated with SE sites in a combined set of all 34 cell types were mapped into 40×25 nodes in a self organizing map and node weight vectors were derived from normalized values of the original variables used to generate the SOM, resulting in six clusters. **b** The names of cell types in each of the six clusters identified by SOM. **c** A comparison of the H3K79me2 enrichment of SE sites vs that of a set of non-SE sites for each of the six clusters

Since SE is the predominant splicing event and is highly enriched for the H3K79me2 mark, we sought to further characterize the genes or transcripts associated with SE sites to dissect their relationship with cancer cell type specificity. We ranked cell type by H3K79me2 enrichment/kb (see the definition in “Methods”) based on the H3K79me2 enrichment level for a total of 7017 genes associated with ES sites in all 34 cell types. Interestingly, we found that two blood cancer cell lines, MV-4-11 and OCI-LY3, have the highest levels. We then performed SOM clustering on the data with at least 100 iterations and obtained optimized parameters that enabled the assignment of all genes into a 40×25 hexagon matrix and definition of six clusters, A to F (Fig. 3a and Additional file 2**:** Figure S5). Each of the 34 cell types was able to be assigned into one of the six clusters. We strikingly identified two clusters, A and F, which consisted predominately of cell lines derived from hematological malignancies: cluster A included AML lines MOLM14, MV-4-11, NOMO1, and OCI-LY3 and the chronic myeloid leukemia (CML) line K562; and cluster F included the B-cell non-Hodgkin lymphoma line Karpas-422, multiple myeloma line MM.1S, diffuse large B-cell lymphoma line OCI-LY7, acute lymphoblastic leukemia line SEM, and lymphoblastoid line GM12878 (Fig. 3b). We also found that clusters C and D were mainly composed of various normal cell types. Further, we examined the enrichment of H3K79me2 between SE sites and non-SE sites in each of the six clusters. Remarkably, we found cluster A had the most significant enrichment difference (1.792 log2 fold change, *p* value < 0.001) and cluster F the second-most (1.671 log2 fold change and *p* value < 0.01) (Fig. 3c and Additional file 2**:** Figure S6). Our results clearly demonstrate that H3K79me2 enrichment within splice sites was highly correlated with blood cell types, especially for AML and B-cell lymphoma. In particular, we noticed that the cell types in cluster A are mainly MLL-r cell types (MOLM14 and NOMO1 are MLL-AF9 and MV-4-11 is MLL-AF4). However, only SEM in cluster F is of the MLL-r cell type. Interestingly, several recent studies have demonstrated the functional role of DOT1L in the development and progression in MLL-r type leukemia [39, 50]. Together, our data reveal the potential regulatory or functional contribution of epigenetic-mediated splicing events to progression of this particular disease.

### Gene expression, Gene Ontology, and motif analyses of SE-associated genes

**Fig. 4:**
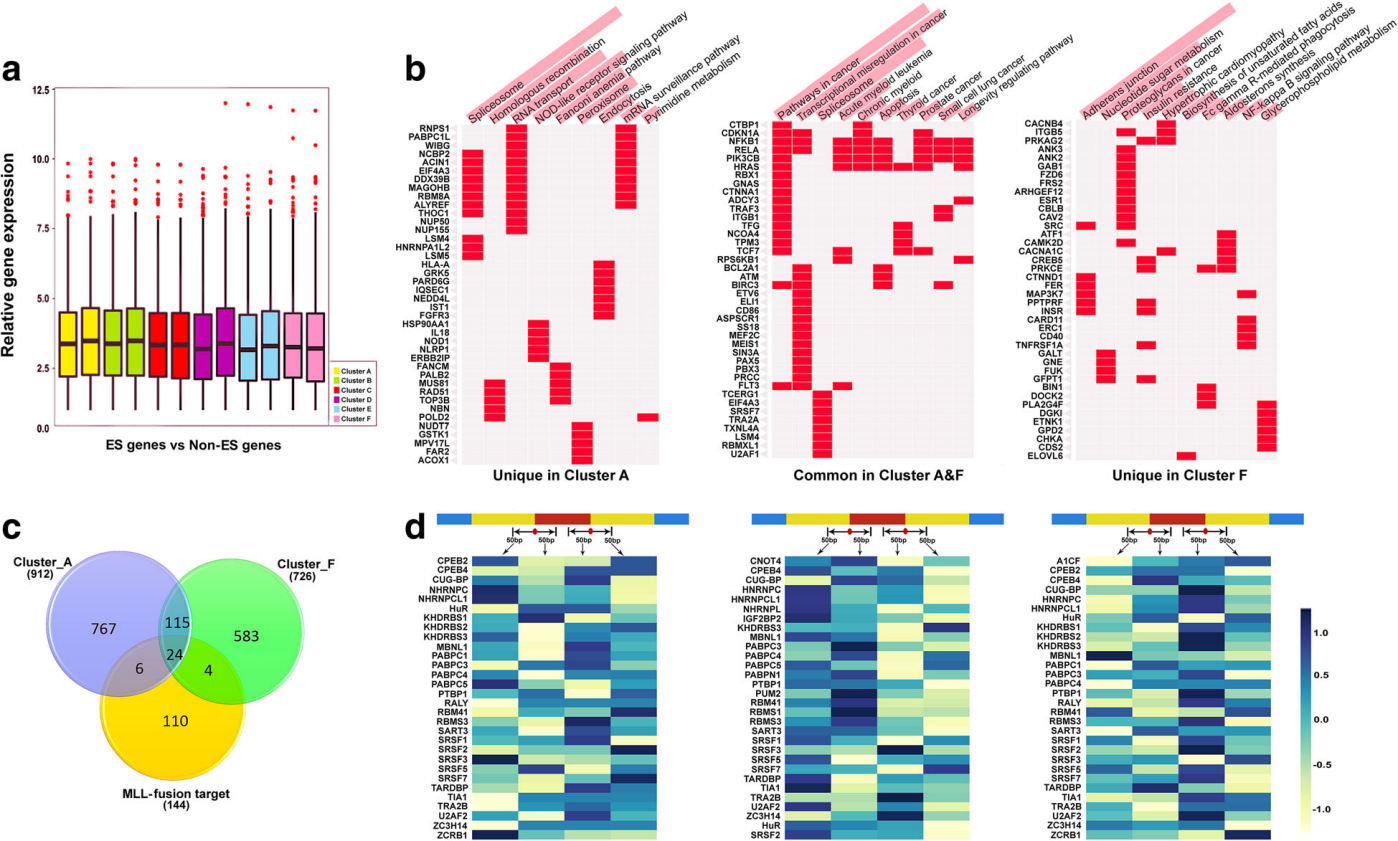
Gene expression, GO, and SF motif analyses of SE-associated genes. **a** A comparison of log2 transformed gene expression levels of genes associated with SE sites vs a set of genes with non-SE sites. **b** Venn diagram showing common and unique genes among clusters A and F and a set of publically available MLL-r target genes. **c** KEGG pathway analysis for unique genes in clusters A (*left*), genes common to clusters A and F (*middle*), and unique genes in cluster F (*right*). **d** Splicing factor motifs identified in each of three sets of genes in **c** at the skipped exon regions

To further examine the expression level of transcripts associated with SE sites, we compared transcripts associated with SE sites with a random set of non-SE genes in each of six clusters and didn’t observe any significant difference between any two sets in any of the six clusters (Fig. 4a). This result is not so surprising given SE sites’ known role in the precursor mRNA (pre-mRNA) regulatory stage as opposed to full mRNA translation [51]. However, it does indicate that the involvement of H3K79me2 in splicing might not impact full mRNA expression. Furthermore, we carried out Gene Ontology (GO) term enrichment analysis using EnrichR [52, 53]. We found that genes in clusters A and F were involved in mRNA splicing via spliceosome with a *p* value < 0.001, as were genes in cluster B (*p* value 0.026). However, genes in clusters C, D, and E were not associated with any splicing events (Additional file 2**:** Figure S7). To further examine the pathway analysis, we overlaid 912 SE genes in cluster A and 726 in cluster F and compared these with a public data set of 114 MLL-r target genes enriched with H3K79me2 [54]. Consequently, we identified 767 unique SE genes in cluster A, 583 in cluster F, and 139 overlapping between clusters A and F, as well as 24 genes common to all three data sets (Fig. 4b). We then carried out KEGG pathway analysis for the unique and overlapping genes in clusters A and F. Interestingly, cancer pathways, transcriptional misregulation, spliceosome, AML, and CML were among the top significantly enriched (*p* value < 0.01) pathways in the overlapping genes between clusters A and F (Fig. 4c).

The *trans*-acting RNA-binding proteins, often called splicing factors (SFs), play central roles in promoting or suppressing the use of a particular splice site. Thus, we searched for SF or RBP motifs in the sequences spanning skipping sites, 50 bp extending into the exon and intron, using the RBPmaptool, a tool designed to map SF binding sites in human genomic regions using the COS(WR) algorithm [55]. We compared the frequency of predicted SF motifs (SFMs) in the four defined genomic regions immediately adjacent to skipping sites versus non-skipping locations. As shown in Fig. 4d, for the common genes in clusters A and F, the top 30 highly enriched SFMs showed a strong tendency towards being within 50 bp of a skipping exon start site, which are highly involved in the exon junction process. In contrast, for the unique genes identified in clusters A or F, the top enriched SFMs were towards the end of the skipped exon. Interestingly, we found that two enriched motifs, SRSF2 and U2AF2, were previously reported to be highly involved in AML progression through aberrant splicing regulation [56] and another motif, PTBP1, was shown to play an important role in breast and colorectal cancers [57, 58]. Taken together, our in silico analyses unveil a potential mechanistic or functional link between H3K79me2-mediated skipping exon processing, splicing factors, and disease progression.

### Functional characterization of DOT1L-mediated SE in MLL-r AML cells

**Fig. 5:**
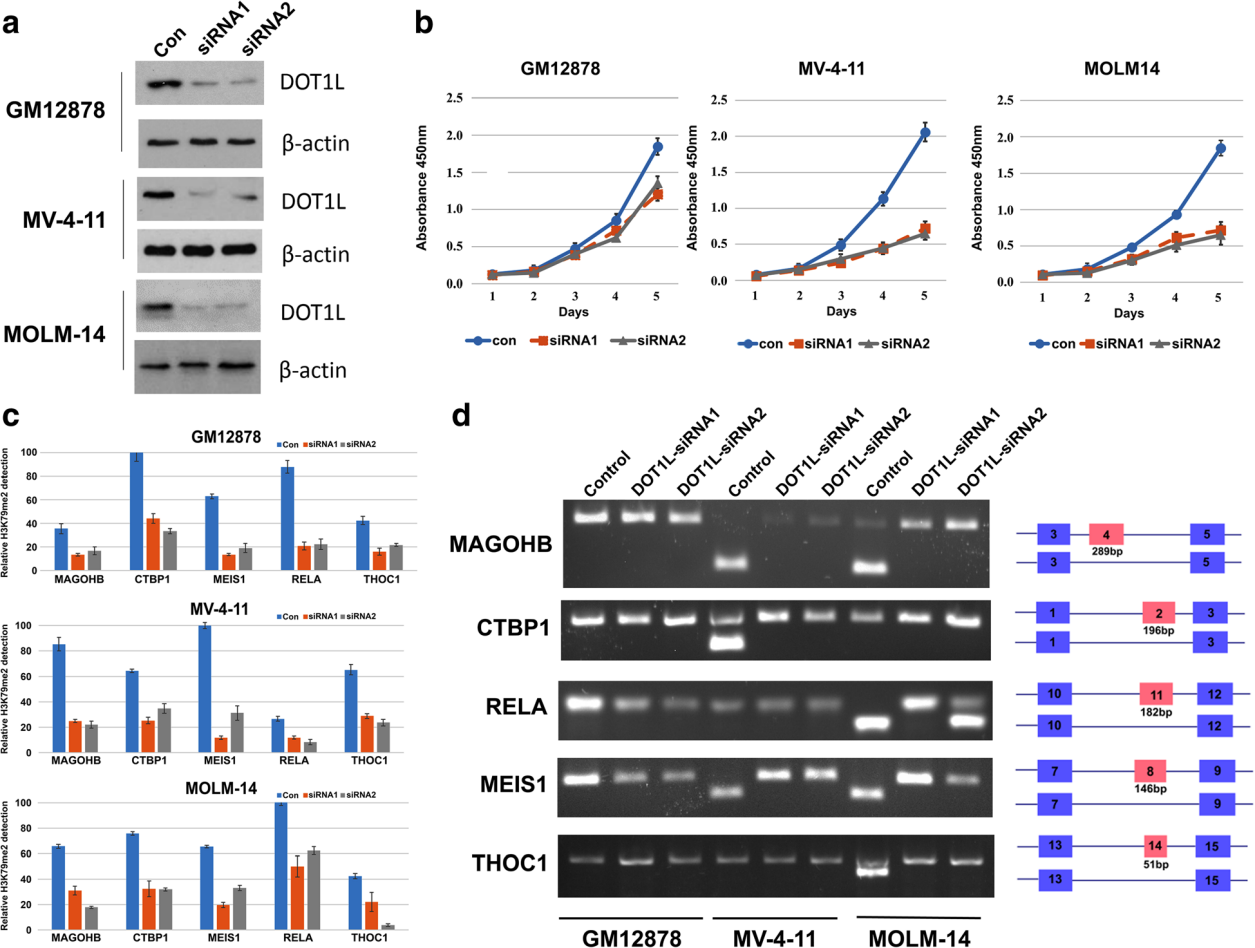
Functional validation of H3K79me2-mediated SE site switching in AML cell lines. **a** DOT1L knockdown by siRNA transfection in GM12878, MV-4-11, and MOLM14 cells. **b** DOT1L knockdown slowing cell proliferation for MV-4-11 and MOLM14 cells but not GM12878 cells. Cell proliferation was evaluated over 4 days of incubation in the CCK8 assay. **c** ChIP-qPCR detection showing reduced H3K79me2 enrichment levels for five specific SE sites after DOT1L knockdown. **d** Exon skipping site switching to the inclusion isoform after the decrease in H3K79me2 levels in five specific gene loci in AML cells

Recent studies have demonstrated that knockdown of DOT1L effectively reduced the H3K79 methylation level in AML cell lines [29, 59] and impeded leukemia cell proliferation [60]. Since DOT1L is the sole K79me methyltransferase, we reasoned that DOT1L is the major regulator in mediating SE in MLL-r AML progression. To functionally characterize the role of H3K79me2 or DOT1L in mediating SE sites in AML, we conducted several functional assays in two selected MLL-r AML cell lines, MV-4-11 (MLL-AF4) and MOLM14 (MLL-AF9), and a lymphoblastoid cell line, GM12878. DOT1L knockdown by siDOT1L clearly reduced the DOT1L protein level in all three cell lines (Fig. 5a) as well as in another three cell lines, K562, MM.1S, and OCI-LY7 (Additional file 2**:** Figure S8). This depletion dramatically impeded proliferation in both AML cell lines (*P* < 0.05 vs control, one-way ANOVA), but not in GM12878 cells (*P* > 0.05) (Fig. 5b and Additional file 2**:** Figure S9). We further tested if this depletion of DOT1L would decrease the enrichment level of H3K79me2. To do this, we selected five genes with exon skipping events, MAGOHB, CTBP1, MEIS1, RELA, and THOC1, from the overlapped genes between clusters A and F and unique genes in cluster A. Indeed, the enrichment of H3K79me2 level at ~ 100 bp around the SE start site was decreased at all five sites in three cell lines after DOT1L knockdown (Fig. 5c). These five genes showed SE events in MOLM14, MV-4-11, K562, MM.1S, and GM12878 cell lines but not in the OCI-LY7 cell line. Strikingly, we found that the exon skipped sites were able to switch to exon inclusion in both DOT1L knockdown AML cell lines, suggesting H3K79me2 is involved in the exon skipping process (Fig. 5d and Additional file 2**:** Figure S10). Although MEIS1 was previously shown to drive MLL-r leukemogenesis through altering DOT1L activity and hypermethylation at H3K79 [39], our data further suggest that DOT1L-mediated splicing drives this leukemogenesis. In addition, we also validated our assertion by re-analyzing public genome-wide datasets. Interestingly, we observed changes in exon usage from SE to non-SE after DOT1L treatment at three concentrations, 58 genes at 0.5 μM, 60 genes at 1 μM, and 73 genes at 2 μM, with an overlap of 14 genes for all treatments (Additional file 2**:** Figure S11) [59]. Collectively, our results support novel regulatory and functional roles of H3K79me2 in mediating AS.

## Discussion

The current paradigm of pre-mRNA splicing centers on a post-transcriptional process mediated by the spliceosome machinery [61]. However, accumulating evidence suggests that AS is a co-transcriptional splicing process not only controlled by RNA-binding splicing factors, but also mediated by epigenetic regulators, such as chromatin structure, nucleosome density, and histone modification [62]. Many recent genome-wide studies, including ours, have revealed the regulatory roles of H3K36me3, H3K79me2, and nucleosome positioning in the RNA polymerase II elongation process and exon definition [18–20]. To further extend our previous study in which we observed a high enrichment of H3K79me2 at skipped exon sites in GM12878 and K562 cells, we conducted an integrative analysis of RNA-seq and H3K79me2 ChIP-seq data across 34 normal and cancer cell types. Intriguingly, we not only confirmed high enrichment of H3K79me2 in SE type splicing events, but also uncovered its enrichment in A3SS events (Fig. 2a). Further, a large proportion of SE sites are characterized by computationally defined H3K79me2 peaks (Fig. 2b), reflecting the high confidence of regulatory activity of H3K79me2 on the exon skipping process.

One novel finding in this study is the identification of six clusters of cell type-specific enrichment of H3K79me2 at skipping exons among 34 cell types. In particular, we discovered that a pattern of histone marks that promote exon skipping was a common feature in cell lines derived from hematological malignancies, in particular MLL-r AML cell types (Fig. 3b). Indeed, when closely examining this in each individual cell type, we observed a clear separation of enrichment of H3K79me2 in a majority of blood-related cell types (Additional file 2**:** Figure S3). Our data highlight the importance of H3K79me2 in AS events across different cell types, but most noticeably in blood cells. Previous studies showed that, for specific gene expression, inactivation of DOT1L led to the downregulation of direct MLL-AF9 targets and an MLL translocation-associated gene expression signature [59, 63]. However, in our study, due to a lack of data for DOT1L inhibition for all 34 cell types, we examined the expression level of transcripts associated with SE sites against a random set of non-SE genes in each cell type and did not observe any significant difference in gene expression between these two sets (Fig. 4a), indicating the correlation of H3K79me2 and SE may be independent of gene expression and such correlation might be through a co-transcriptional pre-RNA splicing mechanism. To our knowledge, this is the first comprehensive study to integrate all available matched RNA-seq and H3K79me2 ChIP-seq data in the same cell type. In a broader aspect, such an integrative strategy may provide a general approach for dissecting the relationship of other histone marks or epigenetic factors with the splicing process and further uncover their novel functionalities associated with various diseases or cancer types, providing a rationale to further explore the underlying mechanism for AML patients without mutations or independent of gene expression.

The gene enrichment and pathway analyses further revealed that H3K79me2-mediated exon skipping-associated genes were highly involved in acute or chronic myeloid leukemia cell types, underscoring their functional relevance to blood cancer progression. Indeed, the various functional assays in this study confirmed that such exon skipping events were highly coordinated by the H3K79me2 or DOT1L activities with DOT1L siRNA treatment in two MLL-r AML cell lines (Fig. 5), providing a new line of evidence of a co-transcriptional splicing process involved in AML. Other studies have shown that higher levels of H3K79me2 are associated with poorer prognosis in MLL-r leukemias [63], and the fusion of DOT1L and MLL partners, AF4, AF9, ENL, and AF10, leads to misregulation of DOT1L targets, resulting in aberrant H3K79me2 activity followed by leukemic transformation [64, 65]. However, our results further unveiled new regulatory and functional roles of H3K79me2 in determining transcript isoforms, providing a mechanistic link between H3K79me2 or DOT1L and splicing events in this particular disease progression.

Our findings may provide a new avenue and opportunity to develop novel combinatorial therapeutic drugs targeting both epigenetic mechanisms and splicing processes. EPZ-5676, a small-molecule inhibitor of DOT1L, is currently under clinical investigation for acute leukemias harboring rearrangements of the MLL gene. Although the agent effectively targets the DOT1L molecule in vitro, the results of a phase 1 clinical trial were disappointing due to low bioavailability and frequent adverse events [39]. In light of this finding, we may consider in future studies testing a co-treatment model which adds the inhibition of a splicing factor as a second synergistic agent, which may enhance efficacy for treating this deadly disease.

## Conclusions

Our study identifies for the first time at a genome-wide scale cell type-specific correlation between H3K79me2 enrichment and skipped exons. This correlation is further utilized to classify the diverse cell types into six distinct clusters. Experimental assays confirm H3K79me2’s functional and regulatory roles in AML disease progression. Our work provides more insights into underlying epigenetic regulatory mechanisms in the co-transcriptional AS process in normal or disease conditions.

## Additional files

Additional file 1: Supplemental **Tables S1–S4.** (PDF 88 kb)

Additional file 2: Supplemental** Figures S1–S11.** (PDF 2762 kb)

## Abbreviations

A3SS: Alternative 3′ splice site
A5SS: Alternative 5′-end splice site
AML: Acute myeloid leukemia
AS: Alternative splicing
DOT1L: DOT1-like histone lysine methyltransferase
MISO: Mixture of isoforms
MLL-r: MLL-rearranged
MXE: Mutually exclusive exon
RI: Retained intron
SE: Skipping exon
SFMs: Splicing factor motifs
SFs: Splicing factors
SOM: Self-organizing map
