# Supplementary material for: Integrative analysis reveals functional and regulatory roles of H3K79me2 in mediating alternative splicing
Source: Genome Med. 2018 Apr 17;10:30. doi: 10.1186/s13073-018-0538-1 (PMC5902843; doi:10.1186/s13073-018-0538-1)
Supplement: Supplementary file 1 — Supplemental Tables S1–S4. (PDF 88 kb) [file 13073_2018_538_MOESM1_ESM.pdf]

Supplementary Information

Integrative analysis reveals functional and regulatory roles of H3K79me2 in mediating alternative splicing

Tianbao Li, Qi Liu, Nick Garza, Steven Kornblau, Victor X Jin

Supplemental Tables

Table S1: RNA-seq and ChIP-seq of H3K79me2 Datasets of 34 cell types.

No.	Cell Type	RNA-seq	ChIP-seq (H3K79me2)
1	Astrocyte	ENCSR233IJT	GSM1003490
2	Primary B cell	ENCSR000CTV	ENCSR051VDI
3	CD14-positive monocyte female	GSM984609	GSM1003537
4	Endothelial of umbilical vein	GSM958738	GSM1003555
5	Fibroblast of arm male adult	ENCSR510QZW	ENCSR611CRY
6	Fibroblast of dermis	ENCFF558ZGC	GSM1003554
7	Fibroblast of lung male ad ult	ENCFF077BBJ	ENCSR000ASF
8	GM12878	ENCFF679NOL	GSM733736
9	H1-hESC	GSM958737	GSM605321
10	HCT116	GSM958749	ENCSR494CCN
11	HeLa-S3	GSM958739	GSM733669
12	Hepatocyte derived from H9	ENCFF464SPX	ENCSR695HYL
13	HepG2	ENCFF928IGY	GSM733641
14	IMR-90 female fetal	ENCSR000CTQ	GSM521911
15	K562	ENCFF696RLQ	GSM733653
16	Karpas-422	ENCFF313CXX	ENCBS301UOD
17	MM.1S	GSM2375013	ENCSR542YRH
18	MOLM13	GSM2136883	GSM2365745
19	Molm14	GSM2236846	GSM2037134
20	MV4-11	GSM1845147	GSM1845135
21	Myotube	ENCFF518DLJ	GSM733727
22	Neural cell	ENCFF921DDO	ENCSR076NBJ
23	Neural progenitor cell	ENCFF433ZEN	ENCSR518LDN
24	NOMO1	GSM1700514	GSM1519632
25	OCI-LY3	GSM1227195	ENCSR957CQH
26	OCI-LY7	ENCFF281CBG	ENCSR347DAG
27	Osteoblast	ENCFF393SFE	GSM1003502
28	SEM	GSM2212245	GSM1934092
29	Skeletal muscle myoblast	GSM958744	GSM733741
30	SK-N-SH	ENCFF154IBY	ENCSR074TRC
31	Smooth muscle cell	ENCFF435NMJ	ENCSR493FIV
32	SUDHL6	GSM1886845	ENCSR471VHW
33	Trophoblast cell embryo	GSM915320	GSM908961
34	LNCaP	GSM721116	GSM918411

Table S2: Primer for RT-PCR detection of Exon-skipping events.

No.	GENE	Exon-skipping region		Skipped exon size
1	MAGOHB	chr12:10762429:10762540:-@chr12:10761694:10761982:-@chr12:10760452:10760535		
		Primer_F:	GCCACAAAGCATCATCTTCTT	
		Primer_R:	GAGCTTGAAATTGTAATTGGAGATG	289bp
2	CTBP1	chr4:1242703:1242743:-@chr4:1235112:1235307:-@chr4:1231970:1232125		
		Primer_F:	TTGAGCAAGTGCGAGCTG	
		Primer_R:	GGACTGCACAGTGGAGATG	196bp
3	RELA	chr11:65423158:65423234:-@chr11:65422290:65422471:-@chr11:65421848:65421980		
		Primer_F:	GAAGCTGAGCTGCGGGAAG	
		Primer_R:	CATTGCGGACATGGACTTCTCA	182bp
4	MEIS1	chr2:66691241:66691352:+@chr2:66739281:66739426:+@chr2:66775074:66775151		
		Primer_F:	CACGGCATCTACTCGTTCAG	
		Primer_R:	TTCACTTGAAGGATGGTGAGTC	146bp
5	THOC1	chr18:225336:225403:-@chr18:225089:225139:-@chr18:224923:224994		
		Primer_F:	GTATCTTCAATCCAAAGTGATTGC	
		Primer_R:	AAACTGGAACTCGTGGAAA	51bp

Table S3: Primer for qPCR detection of DOT1L and H3K79me2 detection for exon-skipping regions.

No.	AS_region	Primer
1	MAGOHB_F	TGATACAAAGTACAATCCTCAGGG
2	MAGOHB_R	TGGGTCTTTTCAACTCTTGGAG
3	CTBP1_F	ACCTGACATCTCTTAATATGGGC
4	CTBP1_R	TCGAGACAATTAAGGACGTGG
5	RELA_F	CACTGCCAACACCCTATCTC
6	RELA_R	GGTGTGGCTAGAACTGGAC
7	MEIS1_F	GATTCGCTATGTTTGCAGGTG
8	MEIS1_R	ACGCCCTCATGATATTTGTGG
9	THOC1_F	CATAGGAGTGGTTTGTAGGCAG
10	THOC1_R	GGATTTTCTTTGTTTCAGCCCC

Table S4: Alternative splicing detection for five types in 34 cell types by MISO algorithm.

No.	34 total	Cancer/Normal	ES	A3SS	A5SS	MXE	RI
1	Astrocyte	Normal	669	212	204	239	170
2	Primary B cell	Normal	984	163	210	276	131
3	Monocyte	Normal	1222	181	193	116	249
4	Endothelial of umbilical vein	Normal	723	129	112	136	110
5	Fibroblast of arm	Normal	467	104	158	215	94
6	Fibroblast of dermis	Normal	1833	169	156	262	81
7	Fibroblast of lung	Normal	1022	342	280	269	128
8	GM12878	Normal lymphocyte	1327	277	172	180	119
9	H1-hESC	Stem cell	741	181	194	171	55
10	HCT116	Colon Cancer	1802	296	260	109	0
11	HeLa-S3	Cervical Cancer	525	115	210	290	123
12	Hepatocyte from H9	Normal liver from Stem	1383	223	136	206	0
13	HepG2	Liver Cancer	592	162	195	143	0
14	IMR-90 fetal	Normal	815	247	224	33	180
15	K562	CML	877	333	286	17	106
16	Karpas-422	B cell non-Hodgkin lymphoma	1260	381	261	207	112
17	MM.1S	Immunoglobulin A Lambda Myeloma	937	247	108	164	128
18	MOLM13	AML	973	192	268	130	143
19	MOLM14	AML	3035	281	118	123	18
20	MV-4-11	AML	1511	234	234	156	0
21	Myotube	Normal from Stem	769	161	227	98	138
22	Neural cell	Normal from Stem	723	219	233	53	107
23	Neural progenitor from H9	Normal from Stem	1248	345	260	58	159
24	NOMO1	AML	1352	201	225	141	175
25	OCI-LY3	B cell lymphoma	1908	0	163	0	124
26	OCI-LY7	B-cell lymphoma	1212	184	301	198	177
27	Osteoblast	Normal	1712	195	294	242	215
28	SEM	B cell precursor leukemia	1833	109	170	28	228
29	Skeletal muscle myoblast	Normal	1962	234	287	123	26
30	SK-N-SH	Neuroblastoma cancer	960	283	245	144	28
31	Smooth muscle from H9	Normal from Stem	575	112	177	238	212
32	SUDHL6	B-cell lymphoma	947	267	187	178	102
33	Trophoblast embryo	Stem cell	2643	153	258	133	41
34	LNCap	Prostate Cancer	330	245	115	24	121
