# Supplementary material for: Integrative analysis reveals functional and regulatory roles of H3K79me2 in mediating alternative splicing
Source: Genome Med. 2018 Apr 17;10:30. doi: 10.1186/s13073-018-0538-1 (PMC5902843; doi:10.1186/s13073-018-0538-1)
Supplement: Supplementary file 2 — Supplemental Figures S1–S11. (PDF 2762 kb) [file 13073_2018_538_MOESM2_ESM.pdf]

Supplementary Figures

Integrative analysis reveals functional and regulatory roles of H3K79me2 in mediating alternative splicing

Tianbao Li, Qi Liu, Nick Garza, Steven Kornblau, Victor X Jin

Supplement Figures

Figure S1: Five types of alternative splicing of exon-junction reads and ψ-value calculation by MISO.

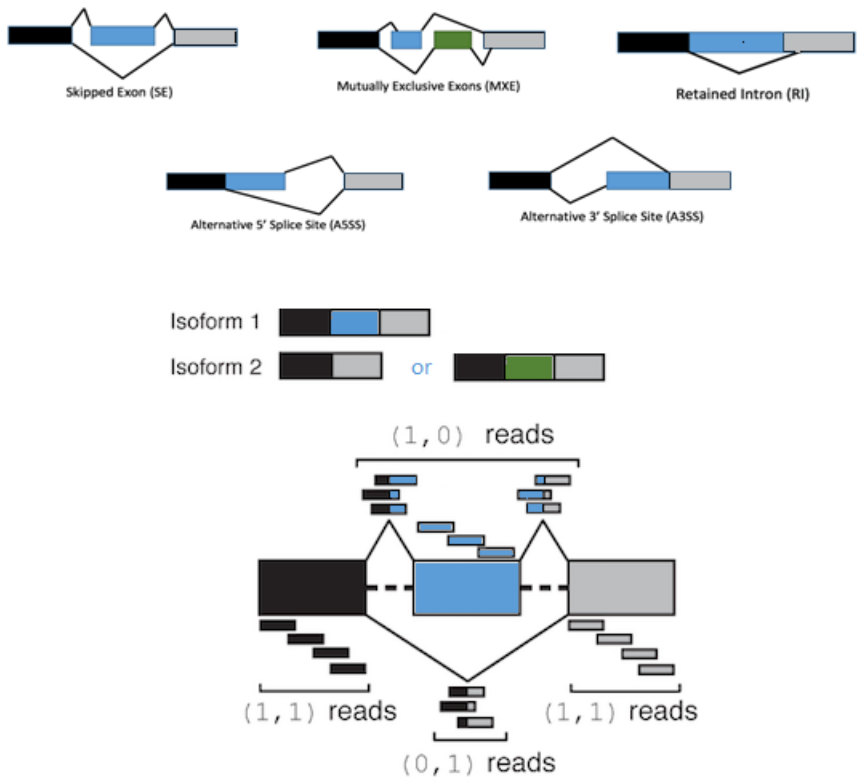

Figure S2: A Circos plot showing the AS events in different loci among 34 cell types

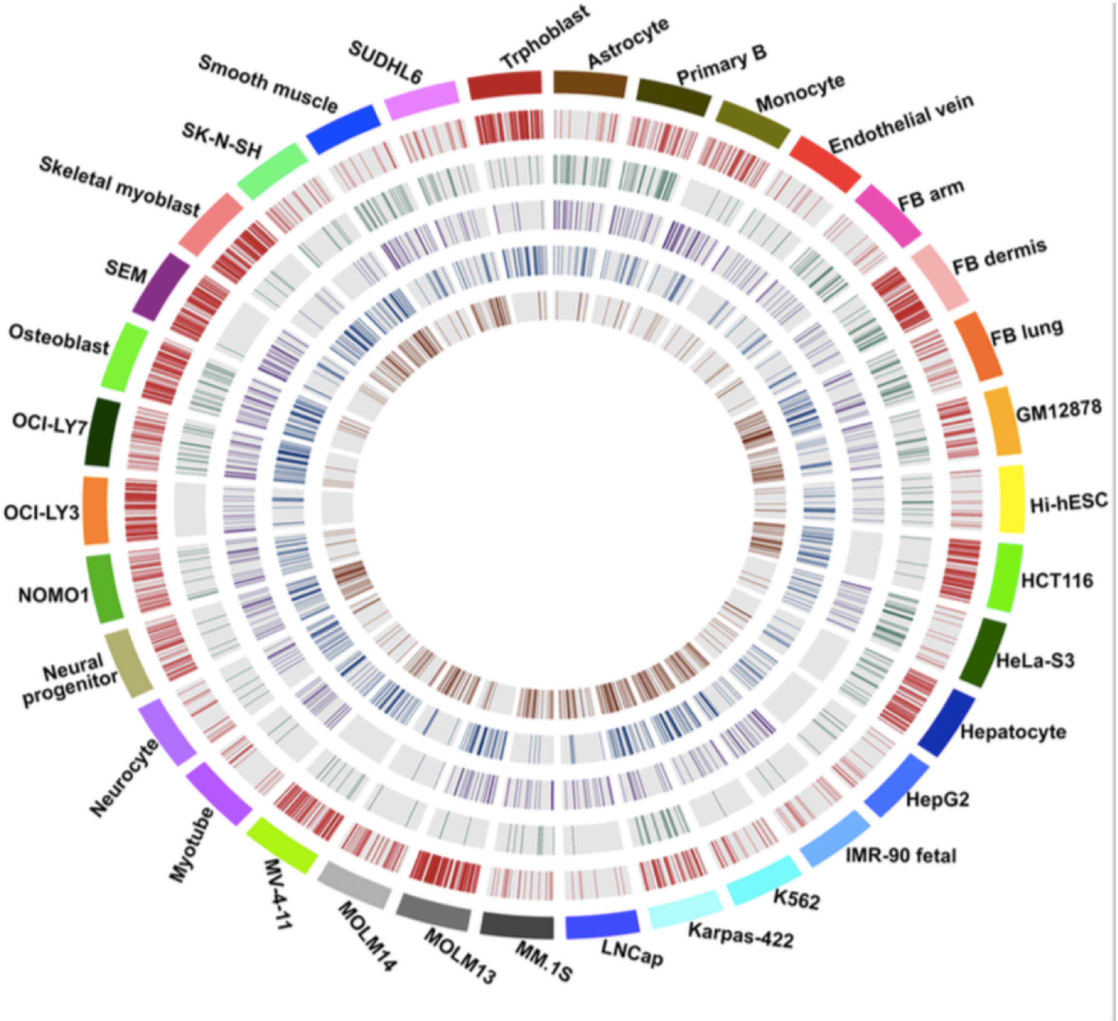

Figure S3: H3K79me2 enrichment around alternative splicing types of MXE, RI and A5SS.

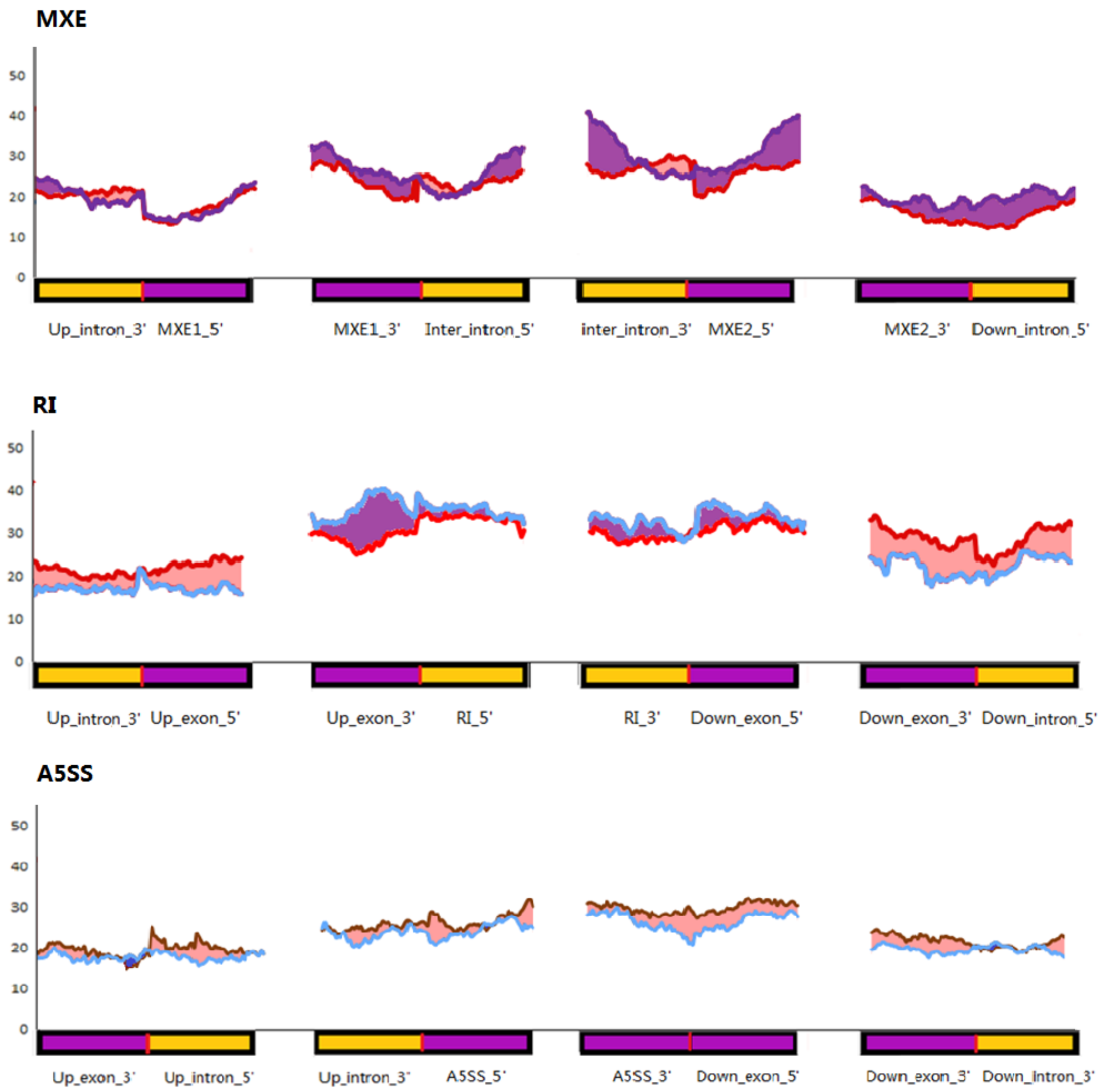

The enrichment levels of H3K79me2 did not show significantly difference in MXE, RI and A5SS splicing types between AS and non-AS regions.

Figure S4: The H3K79me2 enrichment of exon skipping regions in each of 34 cell types.

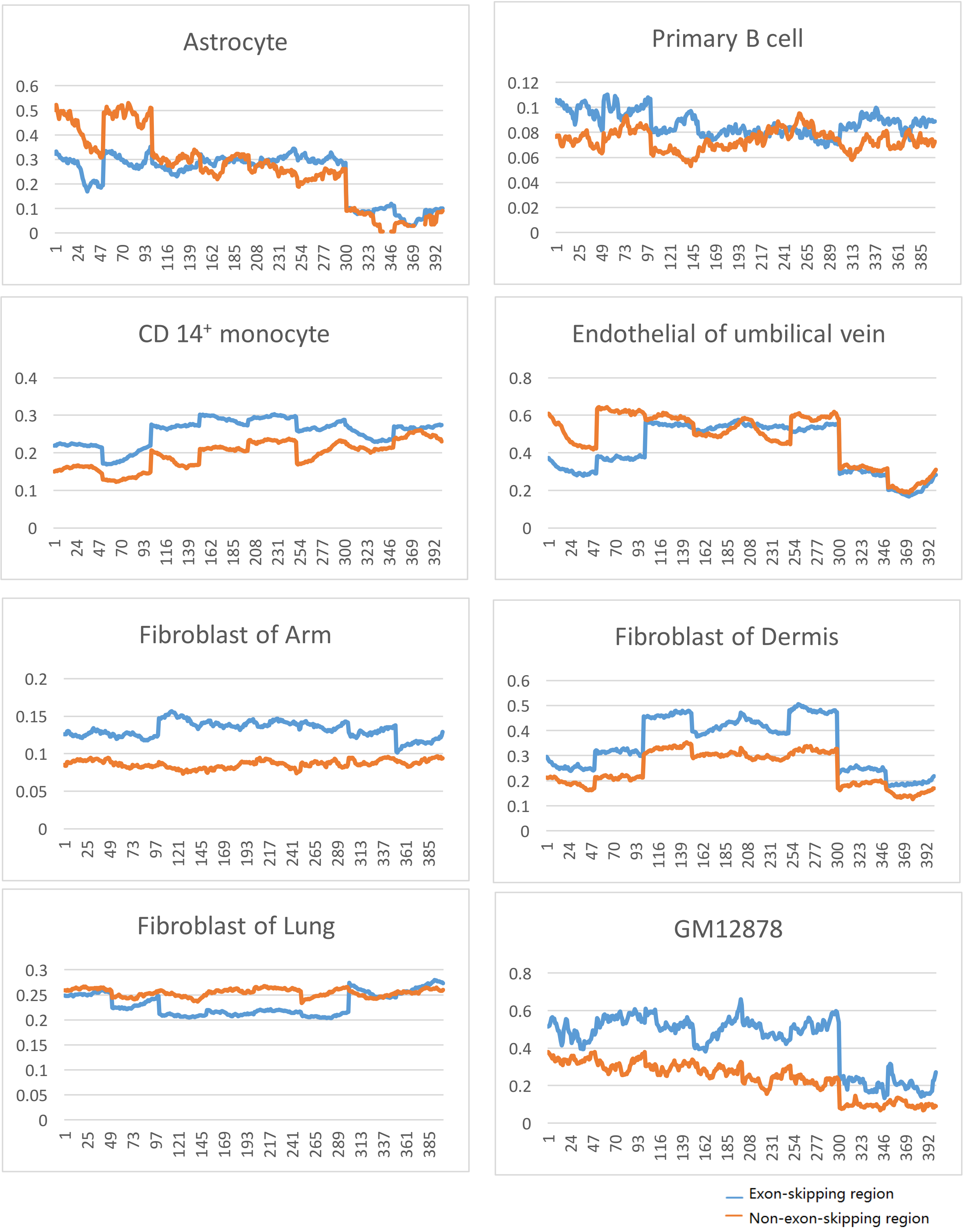

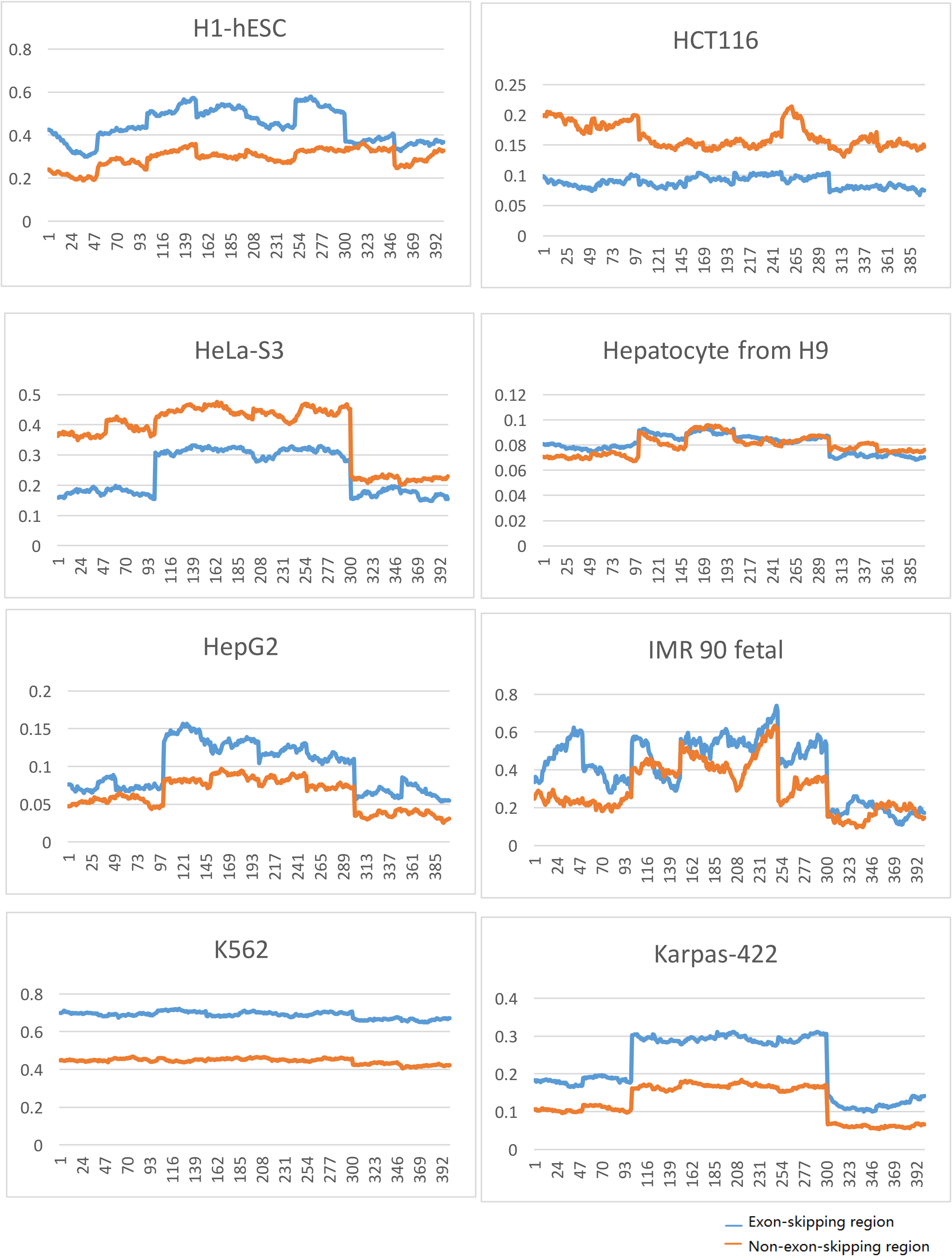
H1-hESC
0.8 0.6 0.4 0.2 0
1 24 47 70 93 116 139 162 185 208 231 254 277 300 323 346 369 392
HCT116
0.25 0.2 0.15 0.1 0.05 0
1 25 49 73 97 121 145 169 193 217 241 265 289 313 337 361 385
HeLa-S3
0.5 0.4 0.3 0.2 0.1 0
1 24 47 70 93 116 139 162 185 208 231 254 277 300 323 346 369 392
Hepatocyte from H9
0.12 0.1 0.08 0.06 0.04 0.02 0
1 25 49 73 97 121 145 169 193 217 241 265 289 313 337 361 385
HepG2
0.2 0.15 0.1 0.05 0
1 25 49 73 97 121 145 169 193 217 241 265 289 313 337 361 385
IMR 90 fetal
0.8 0.6 0.4 0.2 0
1 24 47 70 93 116 139 162 185 208 231 254 277 300 323 346 369 392
K562
0.8 0.6 0.4 0.2 0
1 24 47 70 93 116 139 162 185 208 231 254 277 300 323 346 369 392
Karpas-422
0.4 0.3 0.2 0.1 0
1 24 47 70 93 116 139 162 185 208 231 254 277 300 323 346 369 392
Exon-skipping region
Non-exon-skipping region

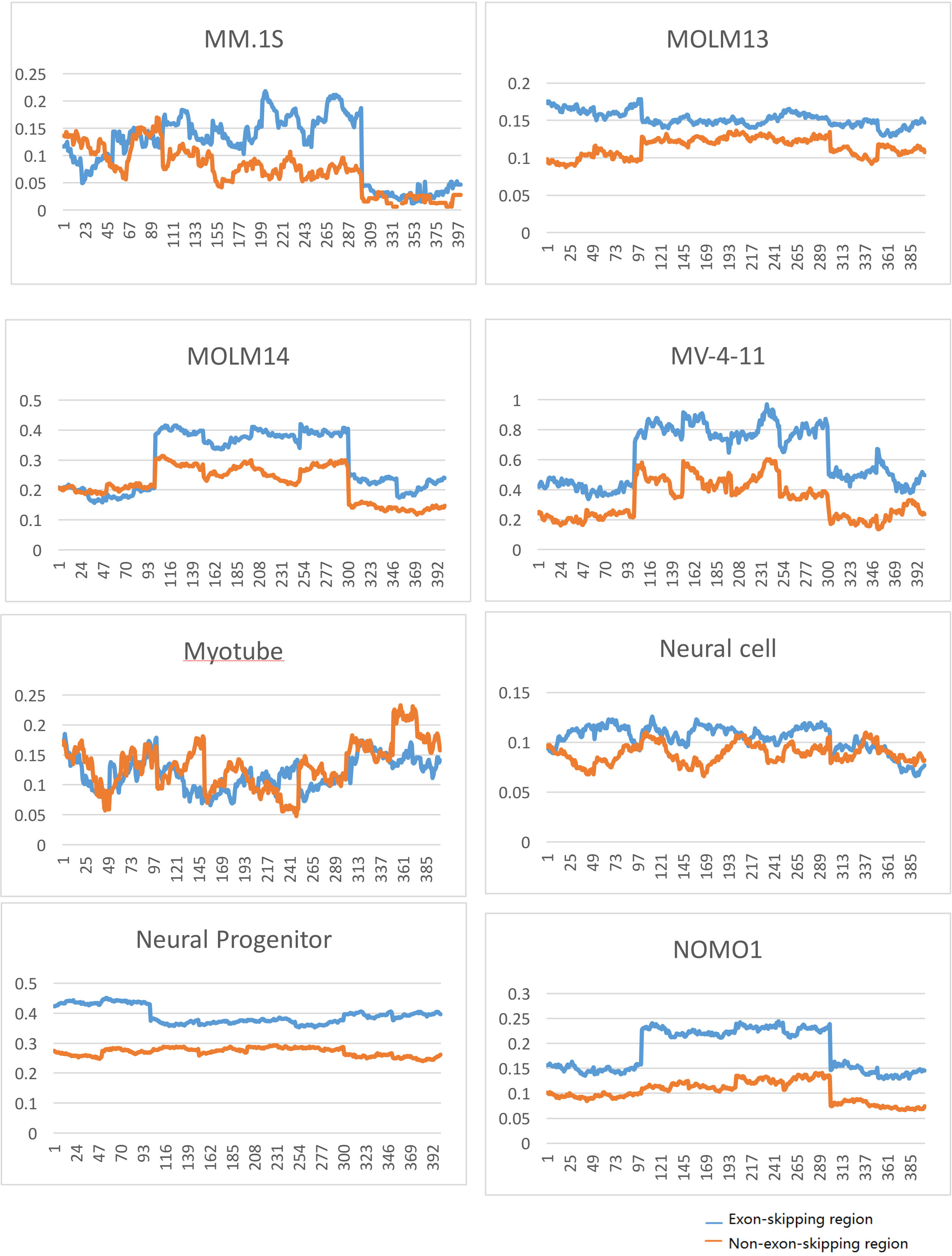
MM.1S
0.25 0.2 0.15 0.1 0.05 0
1 23 45 67 89 111 133 155 177 199 221 243 265 287 309 331 353 375 397
MOLM13
0.2 0.15 0.1 0.05 0
1 25 49 73 97 121 145 169 193 217 241 265 289 313 337 361 385
MOLM14
0.5 0.4 0.3 0.2 0.1 0
1 24 47 70 93 116 139 162 185 208 231 254 277 300 323 346 369 392
MV-4-11
1 0.8 0.6 0.4 0.2 0
1 24 47 70 93 116 139 162 185 208 231 254 277 300 323 346 369 392
Myotube
0.25 0.2 0.15 0.1 0.05 0
1 25 49 73 97 121 145 169 193 217 241 265 289 313 337 361 385
Neural cell
0.15 0.1 0.05 0
1 25 49 73 97 121 145 169 193 217 241 265 289 313 337 361 385
Neural Progenitor
0.5 0.4 0.3 0.2 0.1 0
1 24 47 70 93 116 139 162 185 208 231 254 277 300 323 346 369 392
NOMO1
0.3 0.25 0.2 0.15 0.1 0.05 0
1 25 49 73 97 121 145 169 193 217 241 265 289 313 337 361 385
Exon-skipping region
Non-exon-skipping region

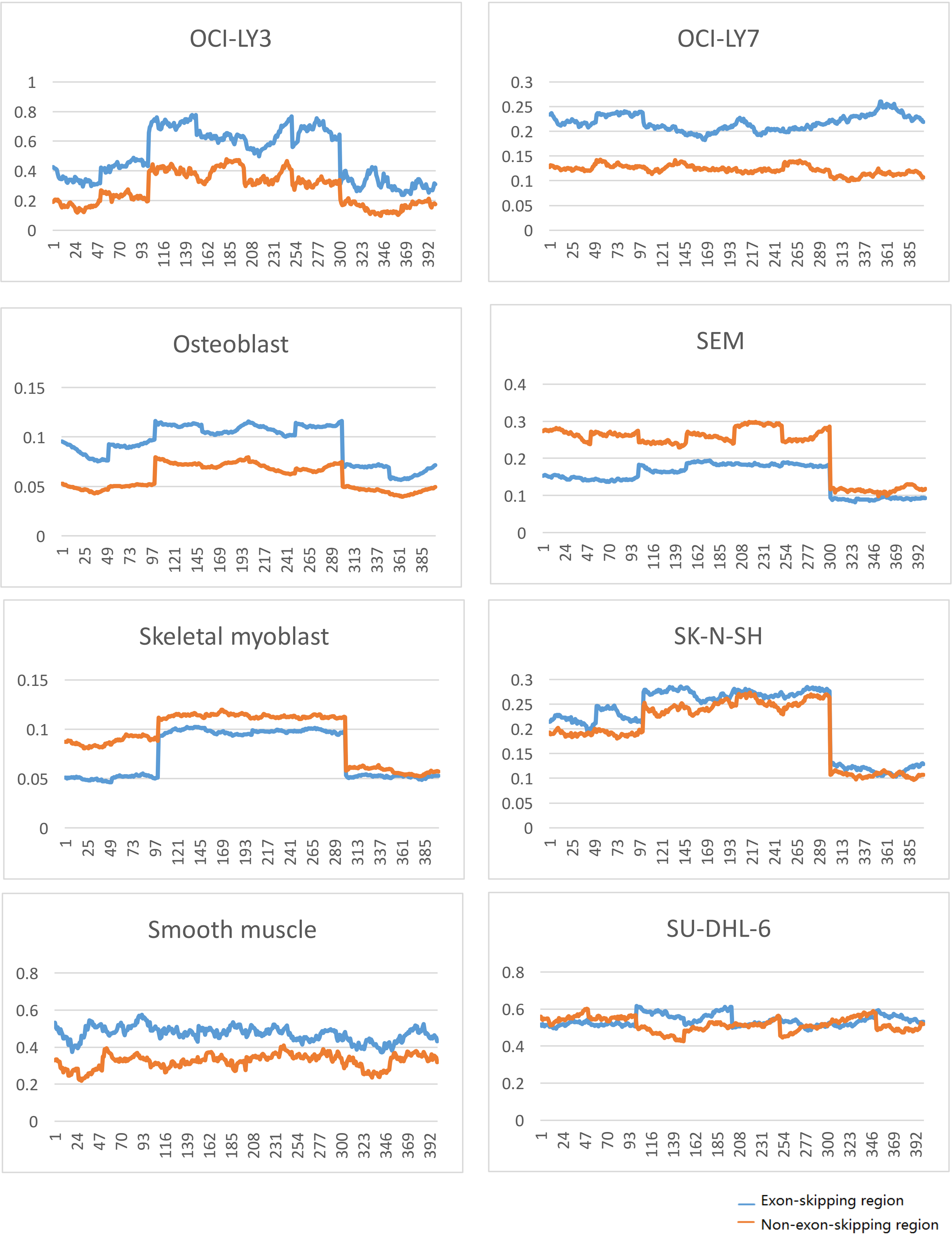
OCI-LY3
1
0.8
0.6
0.4
0.2
0
1 24 47 70 93 116 139 162 185 208 231 254 277 300 323 346 369 392
OCI-LY7
0.3
0.25
0.2
0.15
0.1
0.05
0
1 25 49 73 97 121 145 169 193 217 241 265 289 313 337 361 385
Osteoblast
0.15
0.1
0.05
0
1 25 49 73 97 121 145 169 193 217 241 265 289 313 337 361 385
SEM
0.4
0.3
0.2
0.1
0
1 24 47 70 93 116 139 162 185 208 231 254 277 300 323 346 369 392
Skeletal myoblast
0.15
0.1
0.05
0
1 25 49 73 97 121 145 169 193 217 241 265 289 313 337 361 385
SK-N-SH
0.3
0.25
0.2
0.15
0.1
0.05
0
1 25 49 73 97 121 145 169 193 217 241 265 289 313 337 361 385
Smooth muscle
0.8
0.6
0.4
0.2
0
1 24 47 70 93 116 139 162 185 208 231 254 277 300 323 346 369 392
SU-DHL-6
0.8
0.6
0.4
0.2
0
1 24 47 70 93 116 139 162 185 208 231 254 277 300 323 346 369 392
Exon-skipping region
Non-exon-skipping region

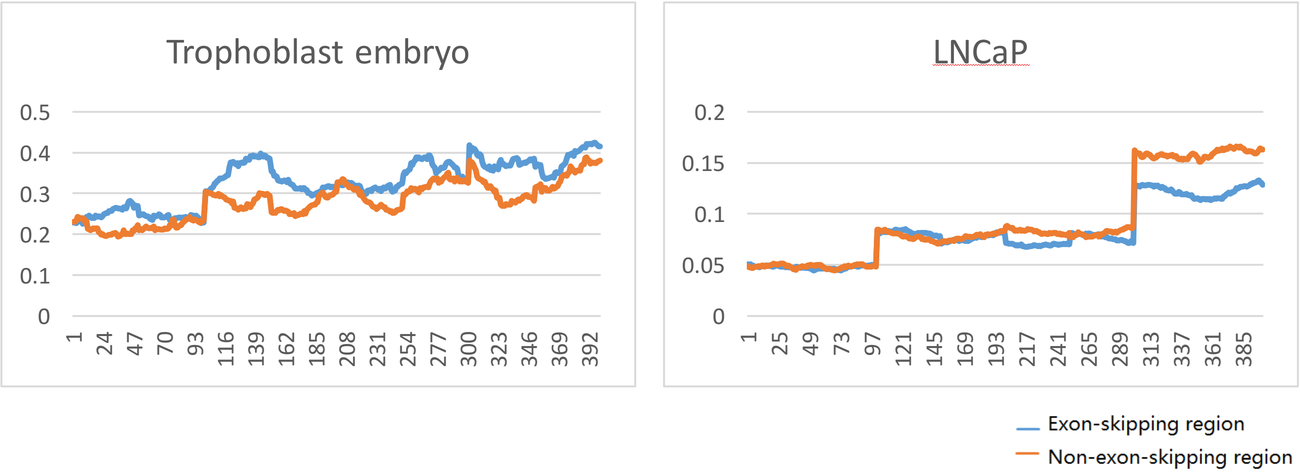
Trophoblast embryo
0.5
0.4
0.3
0.2
0.1
0
1
24
47
70
93
116
139
162
185
208
231
254
277
300
323
346
369
392
LNCaP
0.2
0.15
0.1
0.05
0
1
25
49
73
97
121
145
169
193
217
241
265
289
313
337
361
385
Exon-skipping region
Non-exon-skipping region

Figure S5: SOM clustering optimized process for different winner nodes, weighted vectors and cluster numbers.

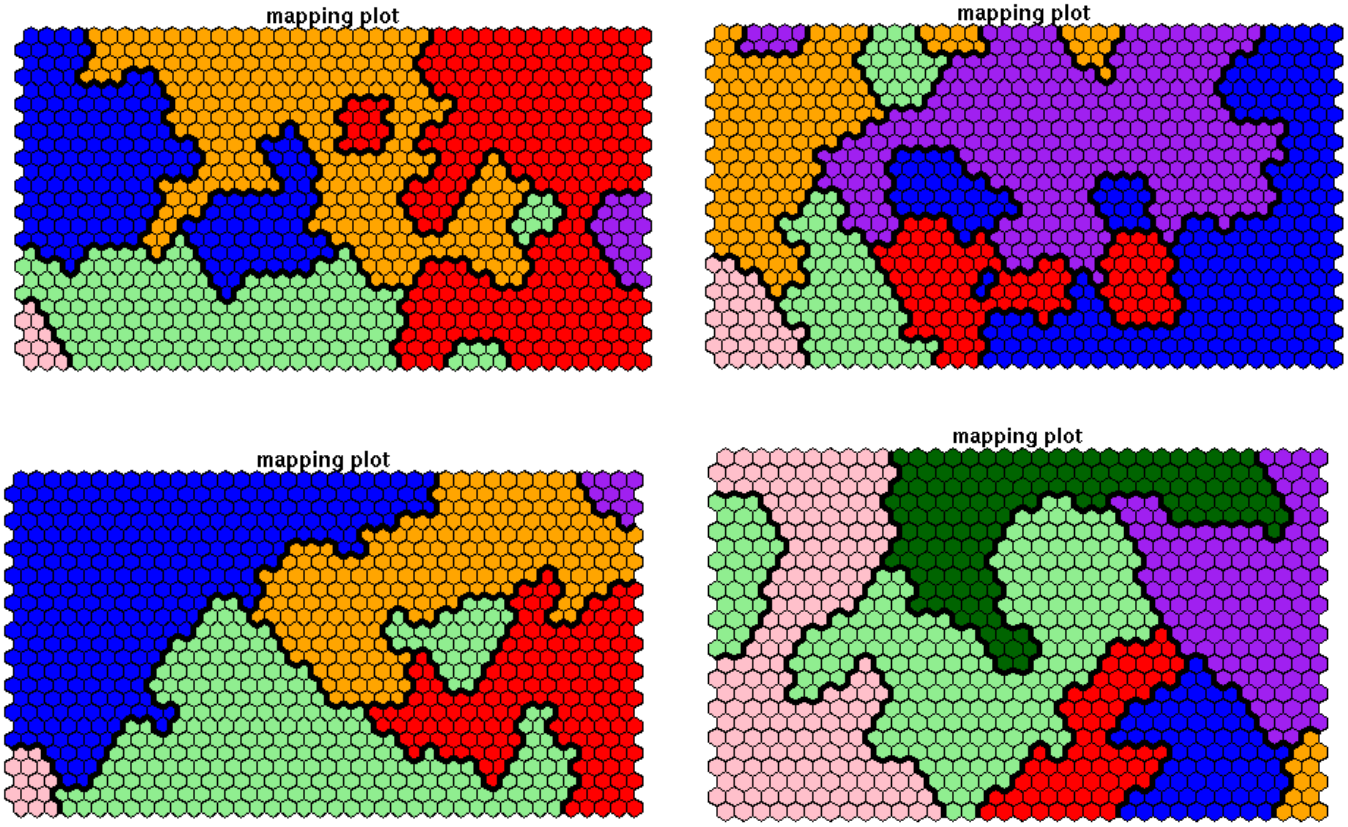

Figure S6: H3K79me2 enrichment level per megabase in different clusters.

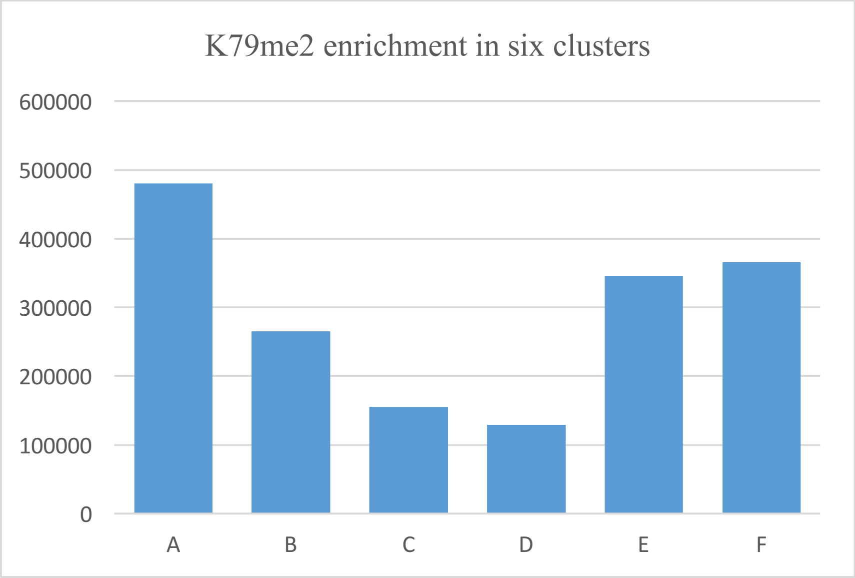

Figure S7: GO analysis of different Clusters shows only Cluster A and Cluster F associated with mRNA splicing via spliceosome.

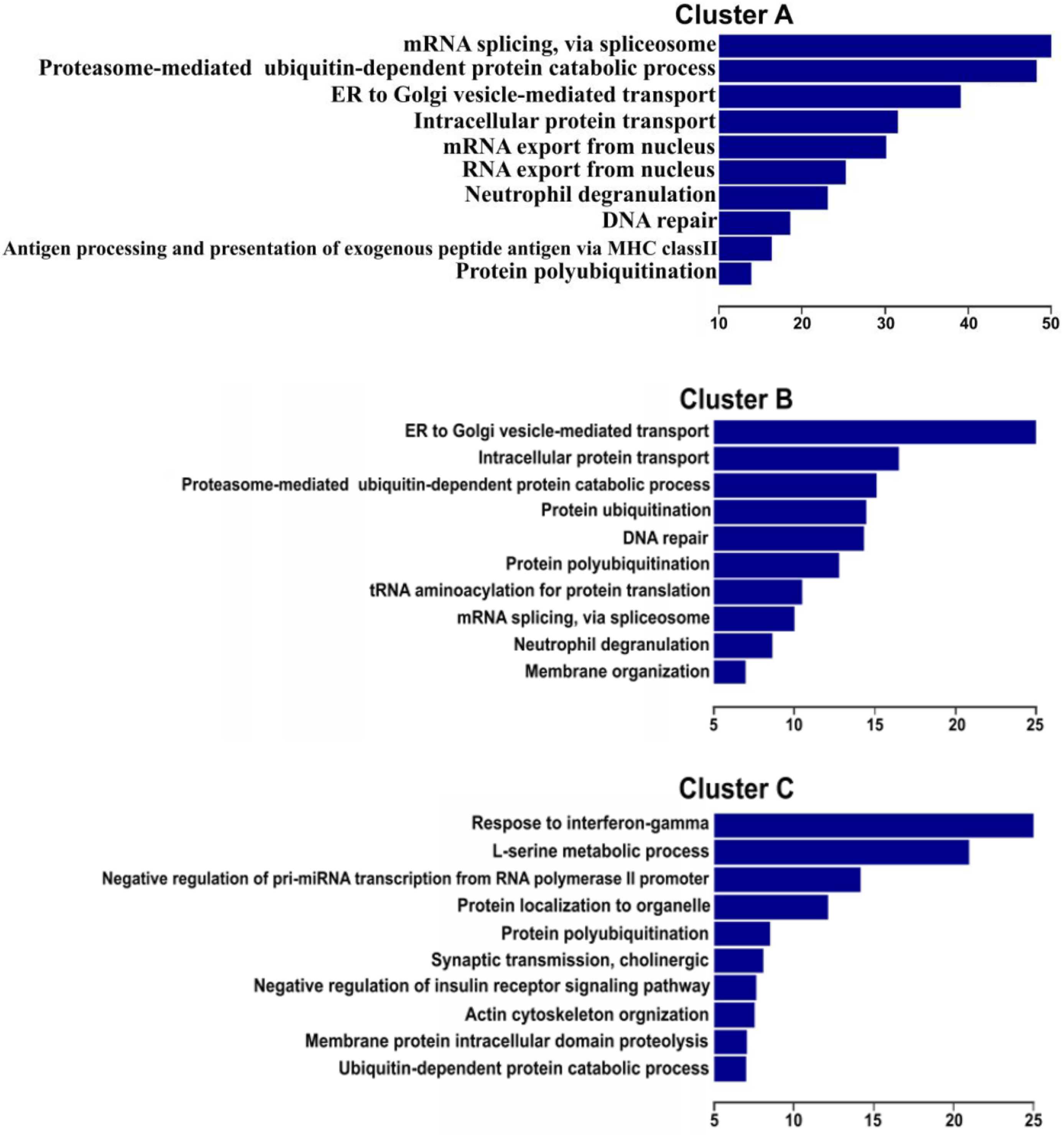

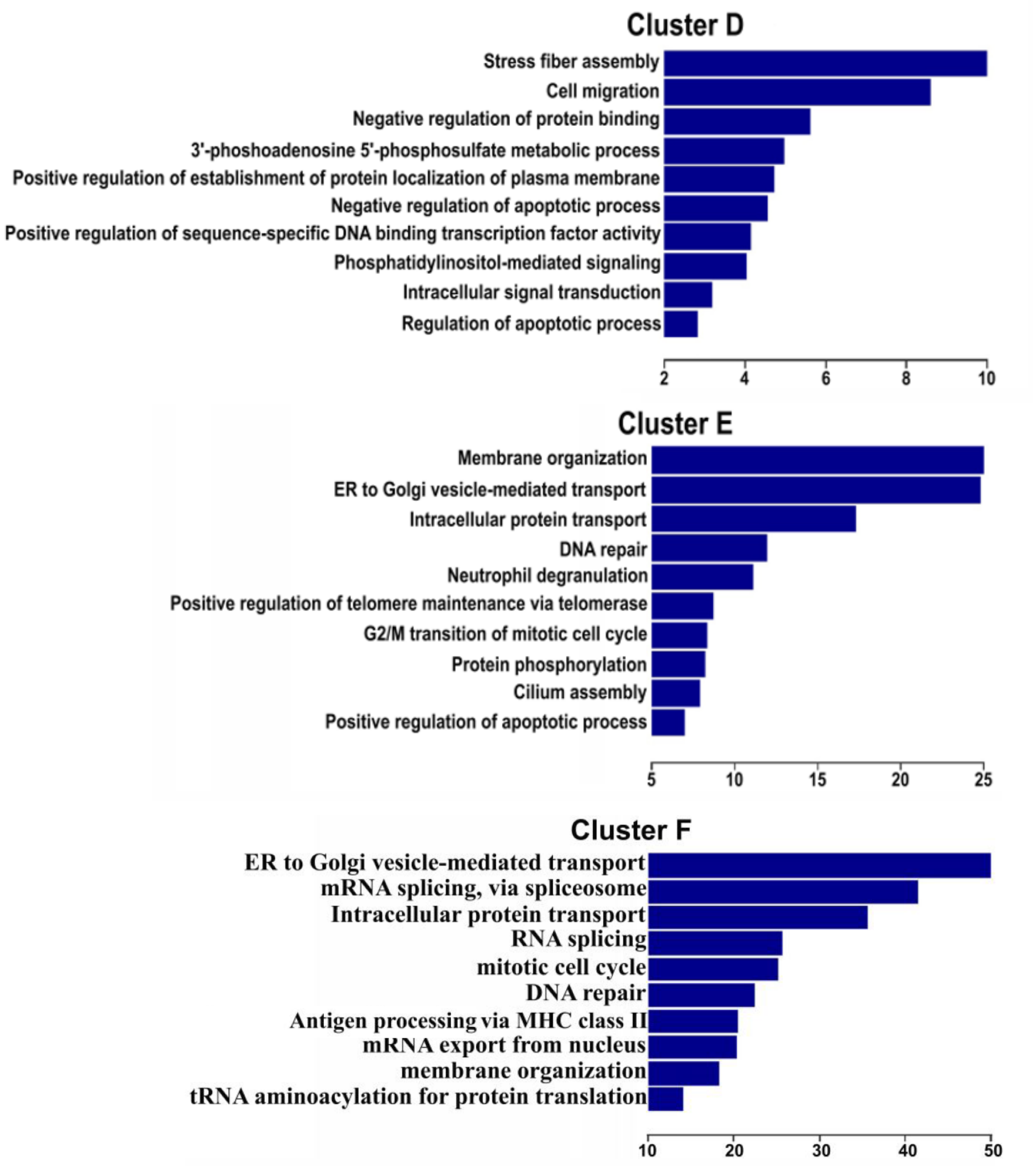

The biology process terms showed that genes from Clusters A and F cells were involved in the mRNA splicing, via spliceosome with a p-value <0.001. Genes from Cluster B were also in the process of mRNA splicing with a p-value of 0.026, however, for genes in Clusters C, D and E, there were not any splicing events.

Figure S8: DOT1L siRNA knockdown effiency detection by qPCR show the expression level of DOT1L descreases in all six cell lines.

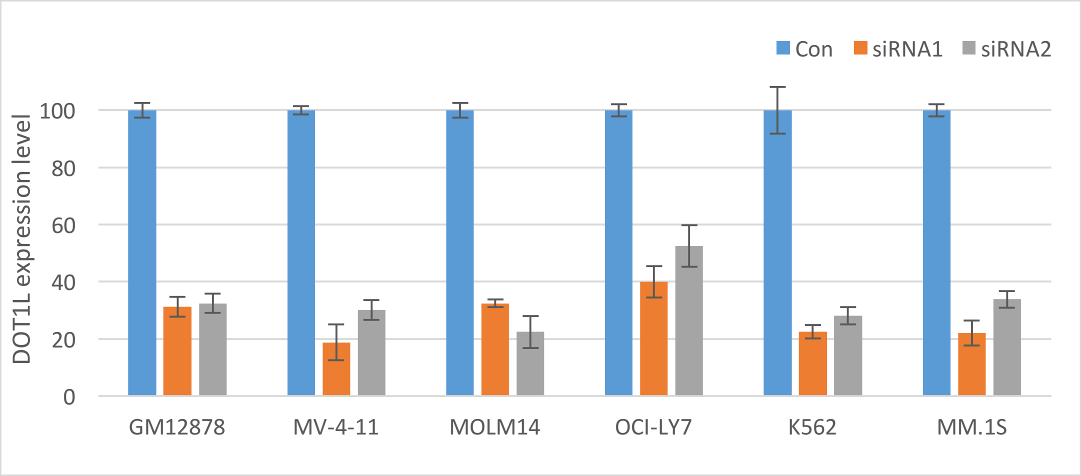

We sucessfully decreased DOT1L mRNA transcription level over 50 percentage in six cell lines with qPCR dectection.

Figure S9: Cell viability was measured by CCK8 assays for 5 days by comparing the control with DOT1L knockdown.

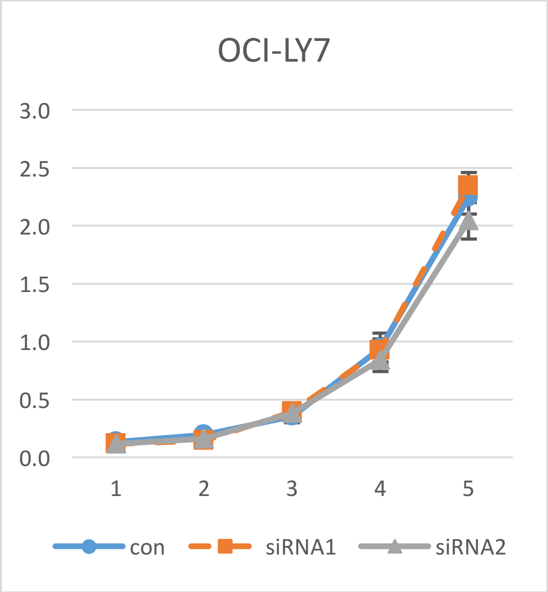

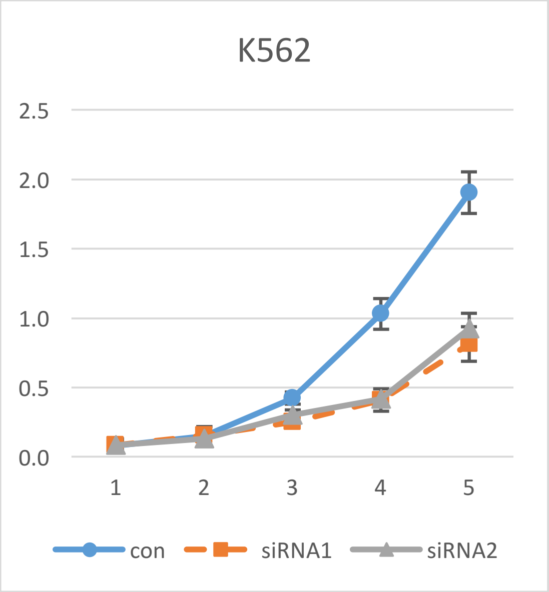

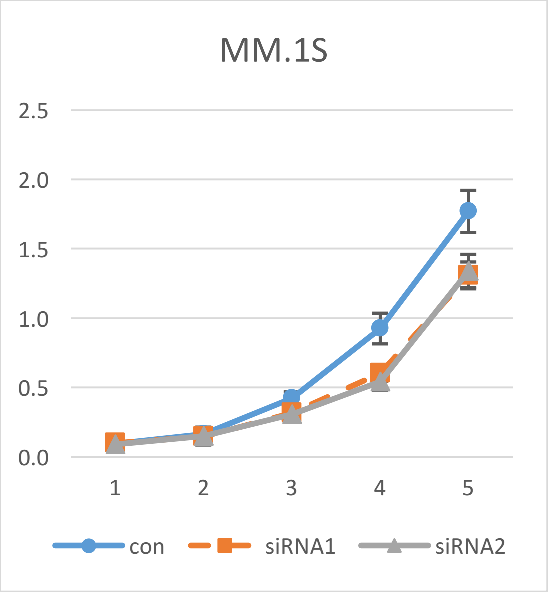

In K562 cells, it showed significant repression of proliferation (p-value<0.05). However, DOT1L knockdown did not affect the proliferation of OCI-LY7 and MM.1S cells.

Figure S10: RT-PCR detection of exon-skipping switching for specific regions upon DOT1L knockdown in three cell lines.

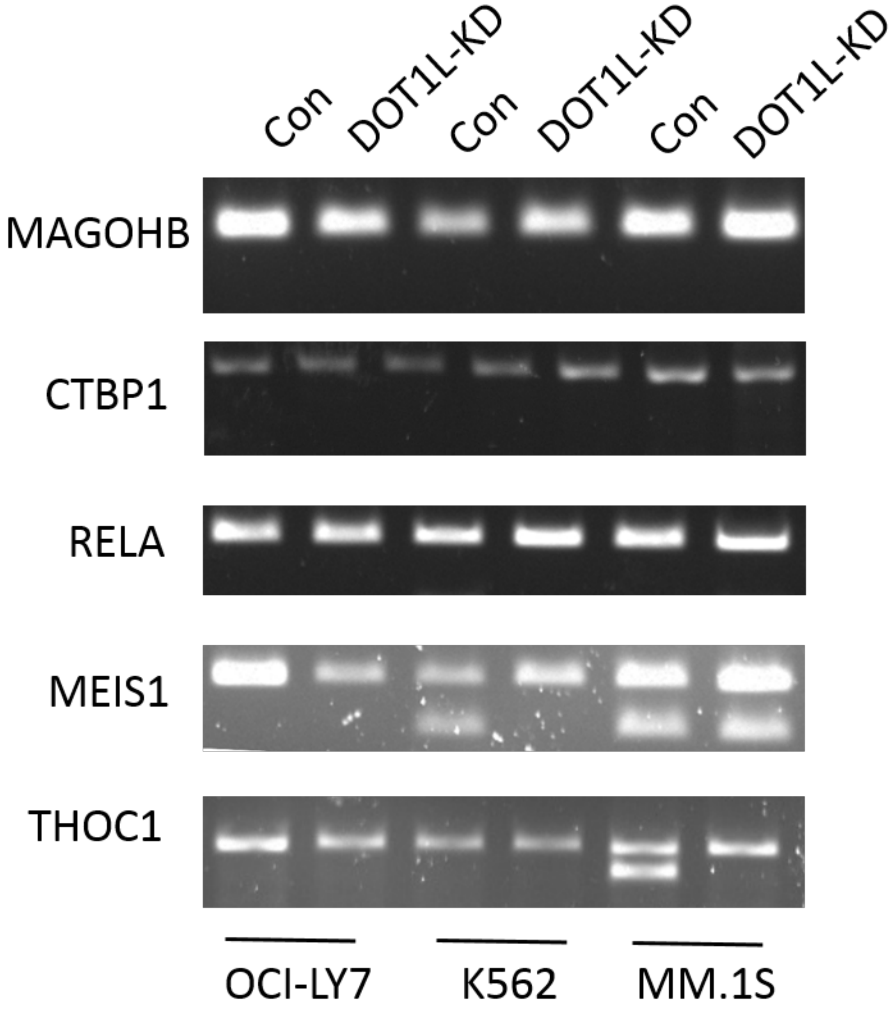

After DOT1L knockdown, MEIS1 exon skipping event was switched in K562 cells, but did not change in MM.1S cells while THOC1 exon skipping event can be restored in MM.1S cells.

Figure S11: Exon skipping detection analysis in publicly DOT1L treated RNA-seq datasets.

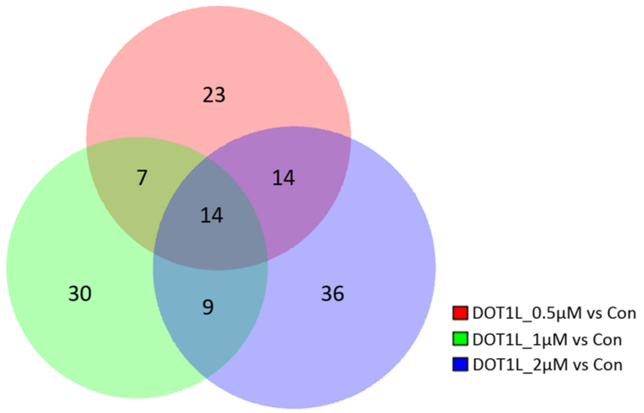

Applying different concentration of DOT1L inhibitor treatment (0.5 μ M, 1 μ M and 2 μ M) comparing with absence of treatment (0 μ M), we did found the changes in exon usage from SE to non-SE after DOT1L treatment (58 genes in 0.5 μ M, 60 genes in 1 μ M, 73 genes genes 2 μ M) and overlap of 14 genes are consistent in all three concentration treatment datasets. The triple overlapping AS genes are as following:

Overlapping	Gene	Psi_con	Psi_0.5uM	Psi_1um	Psi_2um
1	RASSF4	0.15	0.86	0.83	0.87
	chr10:45477966:45478109:-@chr10:45467220:45467296:-@chr10:45465613:45465712:-				
2	SLC20A2	0.19	0.81	0.91	0.86
	chr8:42329622:42330175:+@chr8:42358547:42358975:+@chr8:42396957:42397353:+				
3	FDFT1	0.13	0.8	0.82	0.81
	chr8:11666302:11666401:+@chr8:11667176:11667359:+@chr8:11679259:11679387:+				
4	SPEF2	0.17	0.85	0.81	0.87
	chr5:35667177:35667363:+@chr5:35670159:35670332:+@chr5:35691139:35691360:+				
5	TK2	0.18	0.86	0.87	0.8
	chr16:66562897:66562970:-@chr16:66551693:66551781:-@chr16:66547634:66547714:-				
6	CUL3	0.18	0.85	0.88	0.91
	chr2:225449683:225450111:-@chr2:225422378:225422570:-@chr2:225400245:225400358:-				
7	GLIS1	0.13	0.8	0.86	0.8
	chr1:54199578:54199879:-@chr1:54065772:54065951:-@chr1:54059776:54060662:-				
8	MED13	0.18	0.88	0.87	0.86
	chr17:60112824:60112969:-@chr17:60111148:60111345:-@chr17:60108804:60108999:-				
9	C7orf60	0.16	0.83	0.86	0.8
	chr7:112579643:112579931:-@chr7:112575242:112575319:-@chr7:112555361:112555500:-				
10	ARHGEF1	0.14	0.83	0.88	0.88

	chr19:42410091:42410178:+@chr19:42410609:42410773:+@chr19:42410855:42410956:+				
11	SORBS2	0.16	0.83	0.89	0.81
	chr4:186547985:186548173:-@chr4:186544049:186545645:-@chr4:186541219:186541306:-				
12	KLC2	0.17	0.87	0.88	0.86
	chr11:66026065:66026292:+@chr11:66029213:66029443:+@chr11:66029594:66029664:+				
13	KDM2A	0.11	0.82	0.83	0.82
	chr11:66888704:66888830:+@chr11:66917239:66917333:+@chr11:66947550:66947687:+				
14	CPT1A	0.15	0.87	0.82	0.86
	chr11:68542784:68542901:+@chr11:68548107:68548213:+@chr11:68549238:68549430:+				
